# A comparison of multidisciplinary team residential rehabilitation with conventional outpatient care for the treatment of non-arthritic intra-articular hip pain in UK Military personnel – a protocol for a randomised controlled trial

**DOI:** 10.1186/s12891-016-1309-z

**Published:** 2016-11-08

**Authors:** Russell J. Coppack, James L. Bilzon, Andrew K. Wills, Ian M. McCurdie, Laura Partridge, Alastair M. Nicol, Alexander N. Bennett

**Affiliations:** 1 Academic Department of Military Rehabilitation, Defence Medical Rehabilitation Centre (DMRC), Epsom, UK; 2 Department for Health, University of Bath, Bath, UK; 3 School of Clinical Sciences, University of Bristol, Bristol, UK; 4 Centre for Lower Limb Rehabilitation, Defence Medical Rehabilitation Centre (DMRC), Epsom, UK; 5 Leeds Institute of Rheumatic and Musculoskeletal Medicine, University of Leeds, Leeds, UK

**Keywords:** Non-arthritic hip pain, Femoroacetabular impingement, Multidisciplinary team, Residential rehabilitation, Physiotherapy, Military personnel

## Abstract

**Background:**

Non-arthritic hip disorders are defined as abnormalities of the articulating surfaces of the acetabulum and femur before the onset of osteoarthritis, including intra-articular structures such as the acetabular labrum and chondral surfaces. Abnormal femoroacetabular morphology is commonly seen in young men who constitute much of the UK military population. Residential multidisciplinary team (MDT) rehabilitation for patients with musculoskeletal injuries has a long tradition in the UK military, however, there are no studies presenting empirical data on the efficacy of a residential MDT approach compared with individualised conventional outpatient treatment. With no available data, the sustainability of this care pathway has been questioned. The purpose of this randomised controlled trial is to compare the effects of a residential multidisciplinary intervention, to usual outpatient care, on the clinical outcomes of young active adults undergoing treatment for non-arthritic intra-articular hip pain.

**Methods/design:**

The trial will be conducted at the Defence Medical Rehabilitation Centre, Headley Court, UK. One hundred military male participants with clinical indicators of non-arthritic intra-articular hip pain will be randomly allocated to either: (1) 7-day residential multidisciplinary team intervention, *n* = 50; (2) 6-week physiotherapist-led outpatient intervention (conventional care), *n* = 50. Measurements will be taken at baseline, post-treatment (1-week MDT group; 6-weeks physiotherapy group), and 12-weeks. The primary outcome measures are the function in daily living sub-scale of the Copenhagen Hip and Groin Outcome Score (HAGOS), the physical function subscale of the Non-arthritic Hip Score (NAHS), and VAS pain scale. Secondary outcomes include objective measures of physical capacity and general health. An intention-to-treat analysis will be performed using linear and mixed models.

**Discussion:**

This study will be the first to assess the efficacy of intensive MDT rehabilitation, versus conventional outpatient care, for the management of non-arthritic hip pain. The results from this study will add to the evidence-base and inform clinical practice for the management of intra-articular non-arthritic hip pain and femoroacetabular impingement in young active adults.

**Trial registration:**

ISRCTN Reference: ISRCTN 59255714 dated 11-Nov-2015

**Electronic supplementary material:**

The online version of this article (doi:10.1186/s12891-016-1309-z) contains supplementary material, which is available to authorized users.

## Background

Hip and groin pain in adults have been traditionally attributed to osteoarthritis (OA), however, advancements in imaging and arthroscopy have improved health care providers’ understanding of hip disorders that occur before the onset of arthritis [1]. Non-arthritic hip disorders are defined as abnormalities of the articulating surfaces of the acetabulum and femur before the onset of osteoarthritis, including intra-articular structures such as the acetabular labrum and chondral surfaces [2, 3]. Abnormalities of these structures can lead to a continuum of biomechanical changes and extra-articular adaptations that can cause significant hip pain and dysfunction in young adults [3, 4].

Femoroacetabular impingement (FAI) is a common cause of hip symptoms and impaired functional performance in younger active populations [5]. FAI is a mechanical disorder characterised by repetitive contact between the acetabulum and proximal femur, potentially resulting in damage to the hip joint cartilage and labrum [6]. FAI comprises several hip-shape abnormalities, including CAM and pincer morphology, and has been suggested as a risk factor for hip OA [7]. However, the link between FAI and development of hip OA is unclear. Whilst CAM-type FAI has been shown to be strongly associated with fast-progression to end-stage OA [8], the same association has not been demonstrated for pincer-type FAI and early OA [9]. Therefore, whilst intuitively appealing to conclude FAI structural changes lead to joint damage and development of early OA, a causal link remains unproven [10].

### Conservative management of intra-articular hip disorders

To date, patient outcomes after treatment of intra-articular hip disorders have been limited to post-surgical outcomes [11, 12]. Consequently, comprehensive treatment outside of surgery remains ill-defined [1]. Although the success of conservative management for non-arthritic, intra-articular hip pain is inconclusive [10], positive results have been reported in two recent publications [1, 13]. Hunt et al. [1] and Emara et al. [13] reported outcomes of conservative care for patients with clinical indicators of non-arthritic, intra-articular hip pain. Both studies demonstrated significant improvements in pain and physical function from baseline to +1-year follow-up. The observational nature of these studies prevent speculation on the mechanisms underpinning the effectiveness of conservative treatment, however, these are important findings as many experts had previously concluded there was no role for conservative treatment [10].

A recent systematic review found the experimental evidence reporting non-operative treatment for FAI is limited to 5 articles providing a low-level of evidence [10]. Whilst limited the experimental evidence promotes physical therapy led care for FAI. Fundamental to all regimens is an exercise-based programme focused on the core hip musculature, individually tailored strengthening exercises, manual therapy, home-based exercise and education. In addition, the early use of simple analgesia and NSAIDs is promoted. Whilst there is a suggestion that this form of physical therapy and education confer some benefit to patients, the literature is not supported by any randomised controlled trials, and conservative treatment regimens need to be evaluated more extensively and rigorously to determine the true clinical effectiveness [1, 5, 10, 13].

### Structure and process of hip pain rehabilitation in the UK military

Abnormal femoroacetabular morphology is commonly seen in young men comprising much of the UK military population [14]. Residential multidisciplinary team (MDT) rehabilitation for patients with musculoskeletal injuries has a long tradition in the UK military. Rehabilitation most often takes place in two clinical settings; out-patient primary care rehabilitation facilities (PCRF), and specialist in-patient residential centres. Rehabilitation at the PCRF is focussed primarily on physical function led by a physiotherapist, whereas residential centres have access to larger consultant led multidisciplinary teams delivering a broader psychosocial approach to treatment. However, whilst single treatment entities for hip conditions have been the subject of evidence-based examination, there has been little evaluation of group-based MDT management programmes combining single treatments. Aprile et al. [15], showed no statistically significant difference between outpatient/individual versus inpatient/group hip rehabilitation for any validated clinical outcome measure. Angst et al. [16] found comprehensive inpatient team rehabilitation led to statistically and clinically important improvements in pain and function for patients with co-morbid hip pain. However, there are no studies presenting empirical data on the structure and process of non-arthritic hip pain care for a young active military cohort, or whether a broader residential MDT approach provides better care than individualised conventional outpatient treatment.

With no available data, the effectiveness of MDT residential care remains unclear. Therefore, the purpose of this study is to compare the effects of a residential, multidisciplinary intervention, to usual outpatient care, on the clinical outcomes of UK military patients undergoing treatment for non-arthritic intra-articular hip pain. This protocol describes the design and analysis plan for a randomised controlled trial.

## Methods/design

### Study aims and hypothesis

The primary aim of this study is to compare the effects of a 7-day residential multidisciplinary intervention, to usual outpatient care, on pain and physical function in military patients with non-arthritic intra-articular hip pain.

The secondary aims are to assess changes in relevant musculoskeletal variables of treatment including hip range of motion (HROM), walking ability, and postural control during single leg stance. The primary time point is measured at 3-months (12-weeks).

### Primary hypothesis

No studies have compared the effects of MDT residential rehabilitation interventions versus conventional outpatient care for non-arthritic hip pain. However, systematic reviews and RCT’s provide evidence that MDT inpatient rehabilitation programmes are more effective than usual care (moderate evidence) to improve pain, well-being and self-reported function in heterogeneous patient groups with musculoskeletal conditions [17–20]. Therefore, we will test the following primary hypothesis:

i. A 7-day multidisciplinary residential intervention will result in greater improvements in treatment outcomes compared to individualised outpatient treatment in young adults with non-arthritic hip pain.

### Secondary hypotheses

There is evidence from RCT’s showing that a greater number of therapist contacts and higher intensity rehabilitation improves rehabilitation outcomes in patients with musculoskeletal pathology [21, 22]. This suggests a dose-response for treatment/training outcomes [23]. Class-based supervised exercise rehabilitation has also been shown to improve health outcomes compared to a non-supervised home-based group [24]. In our study a residential rehabilitation group will receive a period of intensive, supervised class-based treatment. An outpatient group will undergo a series of regular clinic appointments combined with unsupervised home-based exercise. We will test the following secondary hypothesis:

ii. In patients with non-arthritic hip pain, residential MDT treatment, compared to outpatient physiotherapist led care, will result in a significant:
  Improvement in hip muscle strength (as measured by hand-held dynamometry)
  Increase in hip range-of-motion (as measured by inclinometer)
  Improvement in health related quality-of-life (as determined by the EuroQuol-5D)
  Greater adherence to home-based exercise (as determined by patient self-report diary)

### Design

The study is a superiority parallel-group, randomised controlled trial (RCT) of a 7-day residential, multidisciplinary intervention (MTD programme), and individualised physiotherapy outpatient care (IP programme) with 3-month follow-up. Measurements will be taken at baseline, post-treatment (7-day MDT; 6-weeks IP) and 12-weeks. We considered including a sham control treatment group but felt in a well-motivated young military cohort this could have a negative impact on recruitment. Trial participants, assessors and clinical/research staff will be un-blinded to treatment allocation. The protocol will conform to the CONSORT (Consolidation of Standards of Reporting Trials) guidelines for non-pharmacological interventions [25], and principles of the Declaration of Helsinki [26].

### Setting

The study will be conducted at a specialist UK military rehabilitation centre.

### Ethics

The study has been reviewed and approved by the UK Ministry of Defence (MOD) research ethics committee (study reference protocol 576 dated 01 Nov 2014). Any requirement for protocol modifications will be submitted for authorisation to the MOD research ethics committee.

### Participants

We will recruit 100 male participants aged 18 to 50 years attending the Centre for Lower Limb Rehabilitation injury assessment clinic (Defence Medical Rehabilitation Centre (DMRC), Headley Court, UK) with symptoms of intra-articular non-arthritic hip pain. Eligible participants must meet the criteria detailed in Table 1.

**Table 1:** Study eligibility criteria

Inclusion criteria	
1. Anterior or lateral hip pain for at least 3-months	
2. Clinical signs and symptoms of prearthritic intra-articular hip pathology/FAI diagnosed by a specialist Consultant Physician^a^	
3. Physical examination findings or reproduction of pain in the groin or lateral hip with the log roll, anterior hip impingement test, Thomas test or resisted straight leg-raise test^b^	
4. Sufficient time to keep therapeutic appointments	
5. Aged ≥ 18 years	
6. Male	
Exclusion criteria	
1. Inflammatory arthropathy	
2. Hip infection or tumour	
3. Hip fracture including history of stress fracture	
4. Existing extra-articular hip disorders and/or any other pre-existing hip pathology	
5. Major structural deformity of the hip	
6. Advanced degenerative disease of the hip (Tönnis classification 2–3) [27]	
7. Any physical impairment or co-morbidities (including cardio-vascular disease) precluding the safe participation in the rehabilitation programme and/or assessment procedures	
8. History of congenital/adolescent hip disease	
9. Cortico-steroid or analgesic injection intervention for hip within the previous 30-days	
10. Clinical signs of lumbar spine disease including radiculopathy	
11. Insufficient capacity to provide informed consent	
12. Aged ≥ 50 years	
13. Female	

^a^Consultant diagnostic criteria will include [1] anterior or lateral hip pain for a minimum of 3-months; [2] history of pain worsening with activity, pivoting, hip flexion or weight bearing; [3] pain associated mechanical symptoms including popping, clicking or locking; [4] pain at rest; [5] physical examination findings or reproduction of pain in the groin or lateral hip with the anterior hip impingement test; [6] physical examination findings that exclude the spine and other lower-limb disorders as a potential source of pain and dysfunction; [7] patient self-report of sensations of instability during functional movements (e.g., squatting)

^b^Measurement techniques and positions are described at appendix 10 of the Additional file 1

### Procedure

**Fig. 1:**
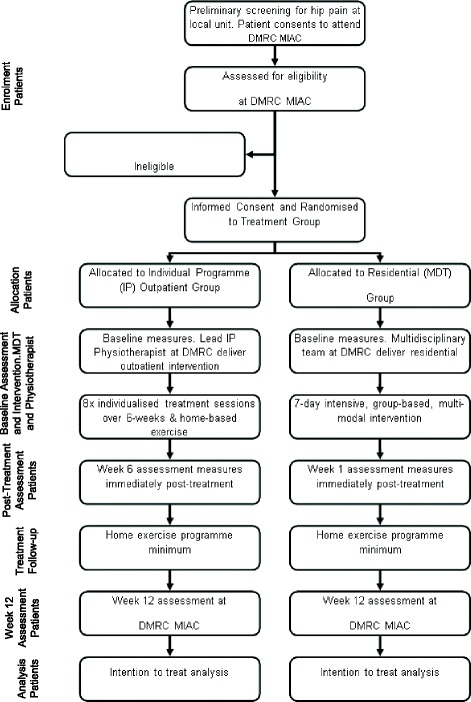
Participant flow through the study. DMRC = Defence Medical Rehabilitation Centre; IP = individualised (outpatient) programme; MDT = multidisciplinary team (residential); MIAC = multidisciplinary injury assessment clinic

The procedure and flow of participants is outlined in Fig. 1. Patients with symptoms of hip pain will undergo preliminary screening conducted by their local unit medical officer. The only screening criteria used at this initial stage is the patient reporting symptoms of hip/groin pain for at least 3-months. Potential participants will then attend a multidisciplinary injury assessment clinic (MIAC) at DMRC for a comprehensive musculoskeletal examination by a specialist rehabilitation consultant, and experienced musculoskeletal physiotherapist. The purpose of the MIAC is to establish the patient’s eligibility for inclusion in the study. Because attendance at the MIAC is directly linked to this study, patient consent to attend the MIAC will be sought in advance via postal consent forms and a patient information sheet, and follow-up telephone calls from study administrators where necessary. The information sheet will detail the nature of the study and will include confirmation that consenting eligible participants will be randomly assigned to one of the following two treatment groups:

i. **Group 1- MDT residential intervention**. Participants will receive an intensive, 7-day residential (in-patient) intervention at DMRC Headley Court. The intervention will include group-based exercise, manual therapy, hydrotherapy and education. The intervention will be delivered by a multidisciplinary team comprising a consultant physician, physiotherapist (PT), occupational therapist (OT) and exercise rehabilitation instructor (ERI). The intervention is anchored by admission and discharge procedures at day one and day seven respectively. Participants will typically complete seven therapy sessions each day of 30–60 mins duration over the remaining 5 days. The sessions enhance social contacts through group-based feedback before and after treatment, and by promoting partner and group exercise [27].
ii. **Group 2- IP outpatient intervention**. Participants will receive 8 treatment sessions, delivered over 6-weeks, combined with a home-based exercise programme. The first and last treatment session will be 45–60 min in duration. The remainder will be 30 min. The intervention will include individualised exercise, manual therapy and individually tailored education/advice. This programme will be delivered as an outpatient service from DMRC and led by an experienced physiotherapist. The treatment session duration reflects a realistic treatment dosage in clinical practice and has been shown sufficient to allow all components of treatment to be carried out [28]. Delivering the intervention in this realistic setting is important to determine the likelihood of effectiveness of the intervention in everyday practice [29]. The home-based programme will initially comprise 3-sessions per week but can be adjusted by the physiotherapist in response to patient progress. The home-based exercise programme in this study will be restricted to 3-times per week based upon the limited available evidence from clinical trials in non-arthritic hip pain [30], and a compromise between addressing the clinical goals whilst optimising adherence [31]. The home-based programme will be tailored to the individual needs of the patient, and utilise self-monitoring by means of an exercise diary.

A definition of rehabilitation based on the concepts of structure and process is frequently used when assessing the content of health care interventions [32]. In our study the therapeutic content (e.g., exercise, manual therapy, education, home-based programme) is the same for both study groups. However, the structure and process will differ significantly. The residential arm is group treatment delivered by a MDT in a residential setting over 7-days. The outpatient arm is physiotherapist led individual treatment delivered during a series of weekly appointments over 6-weeks. Therefore, the amount of direct therapist contact, hands-on physical therapy and instructions surrounding exercise adjustment and education will differ. A detailed description of the treatment methods is contained in Table 3 and Additional file 1.

Eligible patients will be invited to participate in the study and complete an informed consent form. Patients agreeing to participate will be informed of treatment group assignment and referral arrangements by a civilian member of the clinical team, and all necessary study documents will also be provided. Ineligible patients will receive an ongoing referral in accordance with the existing UK military best practice care pathway. Following consent, participants will be consecutively randomised into either the residential or outpatient group by an independent administrator not involved in recruitment, assessment or treatment of participants. Baseline testing will be conducted immediately prior to commencement of treatment. Participants will be reassessed on completion of treatment (1-week (MDT); 6-weeks (IP)), and again at 12-weeks (week 12). During the 12-week follow up period, all participants will be advised to continue with an unsupervised home exercise programme. A log book record of exercise participation will be maintained during this period, and participants will be asked to refrain from seeking other treatments during the study.

### Randomisation and treatment allocation concealment

A block randomisation method will be used to randomise subjects into groups that result in equal sample sizes. This method will ensure a balance in sample size across both groups over time. Blocks are small and balanced with pre-determined group assignments, which keeps the numbers of subjects in each group similar at all times, whilst ensuring there is a 50:50 likelihood of participants being allocated to either group. Due to the possible influence of gender on the efficacy of strengthening exercise [32, 33], the study will only recruit male participants. After patient eligibility has been confirmed and informed consent obtained, a sealed envelope will be opened to reveal group allocation by an independent administrator not involved in the recruitment, treatment or assessment of study outcomes. A plain language statement will inform participants that they have an equal chance of receiving the residential MDT or individualised outpatient intervention. Group allocation will be documented and communicated to the supervising therapists by the independent administrator. Prior to the study, all treating staff will have received a briefing on the study protocol, the ethical, clinical and scientific basis of the study, the randomisation process and specific intervention for each treatment group, in line with the study protocol. It is not possible, relevant or necessary to blind participants to treatment allocation in this study. The clinical staff supervising both groups will be, by necessity, unblinded. Day and Altman [34] suggest blinding assessment by using non-involved assessors to record outcome scores. This is not possible in our study as independent assessors with specific knowledge of quantification of clinical outcomes are not available due to specialist staff man-power limitations. To minimise the potential influence of outcome assessors, instructions are standardised to ensure consistency in measurement. A medical statistician assisting with statistical analysis will be blind to group allocation until completion of the analysis.

### Interventions

The content and protocols for the study interventions are described in a MILO study intervention guide [see Additional file 1]. Table 2 provides a summary of the components of the MDT residential intervention. Table 3 provides an overview of the IP and MDT treatment schedules. Following the recommendations of Bennell et al. [29], a systematic approach to designing all aspects of the intervention has been adopted that ensures it is based on theory, evidence, reflects elements of best practice, and is reproducible and reportable. The MDT residential and IP outpatient group programmes in this study will employ a semi-structured protocol, which provides guidance on exercise prescription, but can be tailored to meet individual patient assessment findings and progression. Written guidelines for individual tailoring of activity and treatment prescription will be provided to supervising therapists. Designing a patient care plan that is individually tailored from a standardised menu of interventions ensures our methodology is consistent with the ‘ideal standard of clinical practice’ [35], and research designs of previous studies [5, 27, 29, 36, 37]. The IP outpatient intervention will be administered by a senior chartered physiotherapist. A broader MDT including specialist physician, physiotherapist, occupational therapist, exercise rehabilitation specialist and nutritionist at DMRC will deliver the residential programme. All treating therapists will attend a training session at DMRC which will focus on the exercise, manual therapy and education interventions to ensure a standardised approach to treatment across both interventions and therapy staff. Whilst the delivery of therapeutic interventions by several MDT staff risks an increase in treatment variation through the influence of therapist personality and style, it allows a more practical delivery mode that mimics clinical practice, and will enhance the generalisibility of study findings [5].

**Table 2:** Multidisciplinary team (MDT) residential intervention – components of treatment

Treatment modality	Treatment content	Treatment goals	Frequency per week (duration)
Group exercise	Strengthening exercises, active range of motion exercises, functional balance drills, gait drills, progressive co-ordination drills, non-weight-bearing aerobic/endurance exercise, minor team games.	Restore strength of deep hip stabilisers, improve core strength, increase joint range of motion, improve balance and neuromotor control, improve muscle endurance, promote group cohesion and social support.	12 (30–45 min)
Individual physiotherapy^a^	Manual therapy techniques, muscle activation and timing patterns, active and passive range of motion exercises, advice on home-exercise, gait re-education training.	Improve quality and timing of movement, improve muscle strength, reduce pain, increase joint range of motion, induce relaxation, promote normal walking gait.	5 (30 min)
Hydrotherapy/swimming	Non weight-bearing aerobic exercise, strengthening exercises, active range of motion exercises, self-paced recreational swimming, progressive/assisted weight-bearing exercise and activity.	Improve muscle strength, improve aerobic capacity, increase joint range of motion, improve confidence in weight-bearing, induce relaxation, promote enjoyment and variety of treatment.	3 (60 min)
Individual occupational therapy^b^	Relaxation techniques, postural re-education, cognitive behavioural therapy (CBT) techniques, self-help coping strategies, pain management.	Induce relaxation, promote behavioural change, control pain, correct/improve poor posture.	3 (60 min)
Patient education	Coping with pain, benefits of exercise, joint protection, anatomy and pathology of hip pain, nutrition.	Activity modification, reduction of pain, promotes behavioural change, weight management, improve knowledge of treatment options, improve ability to relax, improve knowledge of self-help techniques.	2 (60 min)

^a^Exercise dosage, progression and intensity will be governed by the physiotherapist and tailored to the needs of each individual patient; ^b^Occupational therapy referrals will be individually prescribed to selected patients

**Table 3:** Overview of Outpatient Individual Programme (IP Group) and Residential (MDT Group) Study Treatment Schedule

Outpatient (IP) Protocol	Residential (MDT) Programme
Session 1 (45–60 mins)^a^	Day 1
• Subjective and objective assessment (20–25 mins)	• Admission MDT clinic and baseline measures
• Patient education (5-mins)	Day 2
• 1–2 manual therapy techniques (5–10 mins)	• Group-based introduction to treatment goal 1
• Teach target exercise from treatment goals^c^ 1 and 2 (15–20 mins)	• Group-based introduction to treatment goal 2
• Confirm home-exercises (5-min)	• Individual therapy appointments in accordance with patient timetable (PT/OT)^b^
Session 2 (45–60 mins)	Day 3
• Subjective and objective re-assessment (10-mins)	• Group-based introduction to treatment goal 3
• Patient education (5-mins)	• Group-based introduction to treatment goal 4
• Manual therapy techniques (15–20 mins)	• Group-based education topic 1 (‘about hip pain’)
• Teach revised home exercises; check log-book (15–20 mins)	• Individual therapy appointments in accordance with patient timetable (PT/OT)
Sessions 3 to 8 (30–40 mins)	Day 4
• Subjective and objective re-assessment (5-mins)	• Group-based introduction to treatment goal 5
• Manual therapy techniques (15-mins)	• Consolidate individual patient exercise programme
• Patient education & advice (5-mins)	• Group-based education topic 2 (‘activity modification’)
• Progress home-exercises; check log-book; address adherence issues (15-mins)	• Individual therapy appointments in accordance with patient timetable (PT/OT)
	Day 5
	• Group-based exercise targeting individual patient priorities
	• Group-based education topic 3 (‘benefits of exercise’)
	• Individual therapy appointments in accordance with patient timetable (PT/OT)
	Day 6
	• Group-based exercise targeting individual patient priorities
	• Group-based education topic 4 (‘pain management’)
	• Individual therapy appointments in accordance with patient timetable (PT/OT)
	Day 7
	• Confirm individual home-based exercise programme; issue log book;
	• Discharge clinic with multi-disciplinary team
Follow-up period	Follow-up period
• 4 to 6 home exercises, 3-times per week	• 4 to 6 home exercises, 3-times per week

^a^Timing does not include baseline outcome measures; ^b^PT = Physiotherapist; OT = Occupational Therapist; ^c^ Explanation of treatment goals are contained in Additional file 1

The goals of treatment are the same for both the residential and outpatient groups. The specific programme design will differ to reflect the contrasting structure in each setting, and to accommodate the individual participant progression. The core components of treatment are as follows:

### Exercise therapy

It is beyond the scope of this study protocol paper to detail every individual exercise used in the study interventions. The exercise programme incorporates strengthening, flexibility/range of motion, and functional balance and neuromuscular control exercises in accordance with current evidence-based guidelines, and exercise recommendations for the conservative management of intra-articular hip disorders [1, 10, 13].

#### Muscle strengthening exercise

A key component of the exercise programme is local stabilisation of the hip joint by retraining and strengthening of the global hip musculature. We will implement a comprehensive strengthening programme to optimise neuromuscular control, strength and stability of the hip. This includes exercises targeting gluteus medius, gluteus maximus, iliopsoas, quadratus femoris, obturator internus, inferior and superior gemelli, adductor brevis and pectinius [38]. We will also include core strengthening based on studies of hip muscle activity during the performance of core exercises [39], and its recommendation in clinical guidelines for the management of non-arthritic hip pain [40]. We also include functional, weight-bearing hip muscle strengthening including some proprioception exercises to optimise neuromuscular control, stability and strength of the hip in patient specific (military) activities. The programme acknowledges the importance of the deep hip rotators and gluteal group. These muscles act as deep stabilisers to steady the femoral head in the acetabulum, and provide dynamic control of hip joint stability [41]. The focus of the strengthening programme is based on low load exercise, commencing in non-weight bearing positions, 4-point kneeling, and progressing to fully weight-bearing functional positions promoting global muscle recruitment [1, 5]. Three sets of 8–12 repetitions are completed for each exercise. Graduated exercise progression is determined by the supervising therapist based upon participant feedback, re-assessment and individual response to training.

It should be noted that despite an abundance of information on the implementation of strength and conditioning principles with healthy participants, investigation regarding the application of these principles in rehabilitation programmes is lacking [42]. The dosage for strengthening exercises in this protocol aims to meet the ongoing challenge of designing treatment programmes that facilitate neurological and muscular adaptations whilst concurrently accommodating biological healing, recovery and the safety of the patient. The justification for the initial dosage of 3 × 8-12 repetitions takes account of the evidence suggesting pain provoked by exercise has been shown to reduce adherence to exercise in rehabilitation programmes [43, 44]. The study intervention programmes aim to stabilise pain within a given range-of-motion during the early stages of rehabilitation. Therefore a relatively conservative initial dosage is chosen that should allow a short period of adaptation whilst controlling for pain, thereby promoting exercise adherence. This repetition range has been recommended and used for strength exercise prescription in recent RCT protocols/studies for non-arthritic hip pain [5, 30, 45] and hip OA [27, 37, 46]. None provided references to sources of experimental evidence supporting selection of this exercise dosage, however, in two studies [27, 30] improvements in pain and function were reported following this programme.

#### Stretching and range of motion exercise

Decreased range-of-motion (ROM) is common in patients with intra-articular hip pain and osteoarthritis (OA) [47]. Muscle stretching exercises will target the hip flexors, hip extensors, abductors and adductor groups to address and maintain hip extension, flexion, adduction/abduction and internal/external rotation. Static and active stretching and foam-roller techniques are employed to emphasise the ROM needed for activities of daily living. Perception skills and dissociation (joint isolation exercises) are trained to allow proper exercise execution and enhance motor control [27–30]. Retraining optimal control of hip abductor muscles, plus stretching for tight flexor muscles may also contribute to a reduction in destructive joint forces and thereby reduce pain [29].

#### Neuromuscular control and functional balance exercise

Proprioceptive deficits routinely occur in conjunction with articular injuries of the hip [48]. Labral injuries lead to an inhibited motor response and decreased neuromuscular stabilisation of the joint [49]. Balance and proprioceptive exercises will be included to restore these deficits and re-establish neuromotor control. Progression is applied by increasing the complexity and difficulty of the exercise, by reducing the base of support, adding dynamic movements on unstable surfaces, and increasing the range through which the movement is performed. Support for neuromuscular training in hip rehabilitation has been reported in the literature [48].

#### Aerobic exercise

Participants will undertake light to moderate aerobic conditioning over the intervention period. In addition to the general health benefits conferred by aerobic exercise, moderate joint loading has been shown to be beneficial for joint health because of mechanosensitive chondroprotective pathways [50]. No study has described the optimal dose of aerobic exercise for patients undergoing hip rehabilitation in terms of intensity, volume and duration [47]. In this study, the supervising physiotherapist will determine the nature of aerobic exercise (walking, cycling, swimming, cross-trainer), and progression in intensity based on individual examination finings and patient response to exercise. In patients with a differential diagnosis of FAI, static cycling will be undertaken with caution and an elevated seat to avoid combined deep hip flexion and internal rotation [13].

### Manual therapy

Manual therapy techniques will be used to modify the quality and range of motion of the hip and associated soft tissue structures, and assist with pain relief. The manual therapy intervention will be prescribed individually for each participant on the basis of the physical examination findings, from a limited list of techniques including trigger point massage, passive joint mobilisation, distraction and sustained stretches. These techniques are informed by evidence-based best practice and are commonly used in the management of hip dysfunction [5, 30, 36]. Lumbar spine mobilisation, in the form of passive accessory intervertebral movements, will be performed in those patients where the physiotherapy assessment identifies a requirement [5].

### Education

Educating the patient on factors surrounding their treatment and the importance of regular exercise could be a key element for patient adherence to a home-based exercise programme [51, 52]. Education and advice will be a focus of the intervention and will include information on diagnosis and aetiology of FAI/hip pain, rationale for treatment, the benefits of exercise, joint protection and activity modification strategies, pain management, coping with acts of daily living (sitting, driving, sleeping, work), and the importance of increasing physical activity levels in everyday life. The contents of the education component will be evidence-based [10, 40] with the overall focus directed towards increasing the knowledge of the participant on issues surrounding their condition.

### Home-based programme

Participants will be instructed to complete an individualised, unsupervised home exercise programme during the 12-week follow-up period. This programme is prescribed by the physiotherapist at the final treatment session. To optimise adherence, the programme will comprise four to six exercises performed three times per week [29, 30, 35]. The selected exercises will reflect the patient’s clinical priorities for treatment determined by the physiotherapist on completion of the supervised phase of the protocol. The home exercise programme will comprise any combination of strengthening, stretching, neuromuscular control and aerobic exercise. The starting level will reflect the participant’s functional capabilities upon commencement of the follow-up training period. Guidance on exercise progression will also be provided, and participants will receive written instructions demonstrating the home exercises. Participants will be asked to report all training sessions in training diaries. Diary records will be used to measure compliance to the home programme. A goal is that participants should complete a minimum of 80 % of the prescribed exercise sessions [50].

### Outcome measures

Outcome measures with proven validity and reliability have been selected based on those recommended for clinical trials of intra-articular FAI [53], and current practice in UK military rehabilitation [54]. Standardised instruments recommended by OMERACT-OARSI guidelines [55] will also be included. These guidelines require measures of pain, physical function and a global assessment of health related quality of life. Participants will be assessed at baseline, on completion of treatment (1-week MDT group; 6-weeks IP group) and 12-weeks follow up (Fig. 1). The follow-up time-frames are the same for both groups (12-weeks), and all participants will be assessed at baseline and 12-weeks on the same clinical outcome measures. The inclusion of post-treatment measures may help reveal if any differences occur as a consequence of the different interventions (e.g., the immediate post-treatment scores by group). All outcome measures will be conducted, administered and overseen by the same supervising therapist on each occasion. Outcome measures used in the study are summarised in Table 4.

**Table 4:** Summary of outcome measures

Primary outcome measure^a^	Data collection instrument
Function in daily living	Subscale of HAGOS
Physical function, activity level	Subscale of HAGOS
Hip symptoms, numeric pain rating	Subscale of HAGOS, subscale of NAHS, VAS
Secondary outcome measures^a^	
General health status	EuroQol -5D (EQ-5D-3 L)
Mood, anxiety, depression	HADS
Objective functional performance	6-minute walk test
Hip range of motion	Clinical methods and goniometry
Dynamic balance/postural control	Modified Star Excursion (Y-Balance) Test
Hip muscle strength	Hand held dynamometry
Treatment efficacy & self efficacy	SIRBS
Adherence to home-based exercise	Training Diary and 11-point rating scale
Patient demographics & past treatment	Questionnaire

^a^The primary end-point for data analysis is 3-months. All measures will be taken at baseline, post-treatment (1-week MDT group; 6-weeks IP group), 3-months with the exception of patient demographics which will only be assessed at baseline

*HAGOS* Copenhagen Hip and Groin Outcome Score, *NAHS* non-arthritic hip score, *VAS* visual analogue scale, *HADS* hospital anxiety and depression scale, *SIRBS* sports injury rehabilitation beliefs survey

#### Descriptive data

Personal and demographic characteristics including age, height, body mass, body mass index (BMI), gender, duration of symptoms, previous injuries, previous treatments, medication use, military occupation, duration of military service, smoking and drinking habits, and sporting/exercise participation will be obtained by questionnaire.

The primary outcome measures and instruments will be:

- ***The Copenhagen Hip and Groin Outcome Score (HAGOS)***. The Copenhagen Hip and Groin Outcome Score (HAGOS) was developed in 2011 and is specifically designed for young to middle-aged, physically active individuals with hip and groin pain [56]. This patient-reported questionnaire is a quantitative measure of hip disability based on the different levels of the International Classification of Functioning, Disability, & Health. The HAGOS contains 37 questions, covering six domains: pain (10 items), symptoms (7 items), physical function in daily living (5 items), physical function in sport and recreation (8 items), participation in physical activities (2 items), and hip and/or groin-related quality of life (5 items). Construct validity and responsiveness to change have been shown with statistically significant correlation coefficients of 0.37 to 0.73 (*P* < 0.01) for convergent construct validity and from 0.56 to 0.69 (*P* < 0.01) for responsiveness [57]. The HAGOS was selected because it is designed to assess changes from week to week induced by physical therapy [58] in a young to middle-aged population approximating the demographic characteristics of our target sample.
- ***The Non-Arthritic Hip Score (NAHS)***. The non-arthritic hip score (NAHS) comprises 20 questions covering four domains: pain (five items), symptoms (four items), acts of daily living (ADL; five items), and physical activities (six items). The questionnaire was developed for the assessment of hip pain in young participants with increased activity demands [59]. Several questions related to mechanical symptoms and physical activities relevant to active military patients are included. Convergent construct validity has been confirmed with a statistically significant correlation coefficient of 0.82 [57]. The NAHS is currently used within UK military rehabilitation, and scales of this kind are frequently used as an external criterion for comparison with changes in scores of other outcomes [5]. Therefore, the NAHS will be used to assess similarity of results with other self-reported measures included in this study.
- ***The Visual Analogue Pain Scale*** *.* A visual analogue scale (VAS) will be used to measure pain intensity. The patient will be asked to rate their worst hip/groin pain experienced over the past 24-h using a 100 mm horizontal VAS anchored by the terms ‘no pain’ and ‘worst pain possible’. The supervising physiotherapist will also enquire if the sensation of pain is sharp or a dull ache, and if pain is made worse by movement. The 100-point VAS scoring requires no mathematical transformation, and with a normal distribution of data, it allows for parametric statistical analysis [60]. The VAS response format has shown good internal consistency, is easy to understand, is in wide clinical use, and has been sufficiently evaluated in clinical trials [60, 61].

**Fig. 2:**
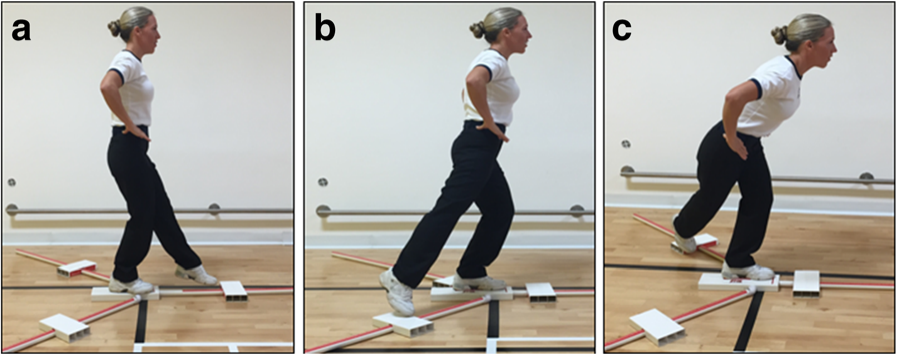
Y-balance test. From a single-leg stance the participant reaches the freely moveable limb along a line in the **a** anterior, **b** posterolateral, and **c** posteromedial directions
- ***Hip Range of Motion (HROM)*** *.* Loss of range of motion is a common clinical finding in hip OA and intra-articular hip disorders, and is associated with pain and disability [1, 2, 36, 40]. Passive range of motion will be measured on both sides for hip flexion, abduction and medial rotation using a high precision inclinometer. Individual scores will be recorded and used for analysis purposes. High test-retest reliability values have been reported (0.82, 0.86, and 0.90 respectively using the intra-class correlation co-efficient (ICC)) in subjects with hip OA [36, 77]. Whilst this is a standard assessment commonly employed in the clinical setting, consistency in measurement technique will be confirmed during a 1 day training session at DMRC prior to commencement of the study. A description of testing positions and techniques is provided in Additional file 1.
- ***Hip Muscle Strength*** *.* Strength assessment plays an important role in the clinical examination of the hip [78, 79]. Muscle strength measurement in this study will be analysed in parallel with the assessment of physical function to establish the contribution changes in muscle strength may have on functional performance. Hip muscle strength will be measured on both sides for flexion (FL), extension (EX), abduction (AB), adduction (AD), internal rotation (IR) and external rotation (ER). Strength measures will be taken using hand-held dynamometry (HHD). Participants will be tested on a clinical examination couch in either a seated or supine position depending on the movement being measured. Test positions were chosen based on procedures often applied in the clinical setting [79–81]. In accordance with the description of Thorborg et al. [78] an isometric ‘make-test’ will be used for testing. This test was chosen as isometric loading induces less stress on the musculoskeletal system than eccentric loading (‘break-test’), which is a key consideration when testing individuals with a physical injury [79]. A long lever arm will be utilised during the six individual tests wherever possible to ensure the tester’s strength exceeds the isometric force applied by the participant. For each movement the examiner will apply resistance in a fixed position whilst the participant exerts a 5-s isometric maximal voluntary contraction (MVC) against the dynamometer and the examiner. Participants will perform four consecutive attempts for each movement with a 30-s recovery between attempts. Strength measures will be reported as Newtons (N). The highest value will be used for analysis purposes. Good interrater reliability (ICC 0.76–0.79) and low test-retest variation (<10 %) has been demonstrated for the HHD measurement technique in measuring hip muscle strength [78, 81]. Descriptions of measurement techniques are provided at appendix 10 in Additional file 1.
- ***Sports Injury Rehabilitation Beliefs Survey (SIRBS)*** *.* The purpose of the education programme in this study is to empower patients to manage their own pain and to maintain physical function. Perceived self-efficacy is defined as a person’s judgement or belief of their ability to change, manage or execute tasks related to pain [82]. Several studies have found that a patient’s perceived self-efficacy is related to health outcomes and the course of disease progression [82, 83]. The Sports Injury Rehabilitation Beliefs Survey (SIRBS) [84] is a 19-item instrument that contains five subscales assessing severity, susceptibility (threat appraisals), treatment efficacy, and self-efficacy (coping appraisals). Ratings are made on a 7-point Likert-type scale ranging from 1 (very strongly disagree) to 7 (very strongly agree). In this study, only the treatment efficacy (4-items), and self-efficacy (4-items) subscales will be used. Acceptable alpha coefficients have been reported for treatment efficacy (0.85) and self-efficacy (0.91) [85], and low-to-moderate interscale correlations provide some support for its construct validity [84].
- ***Adherence to Home Exercise*** *.* Low adherence to home based exercise programmes is widely accepted as a key contributor to poor long-term clinical rehabilitation outcomes [85, 86]. To measure adherence during the 3-month follow-up period, participants in this study will be asked to maintain a diary/log-book recording the frequency, duration and intensity of the exercises in their home-based programme. Participants will also rate their adherence to the home programme at 3-months on an 11 point rating scale (with 0 being ‘not at all’ and 10 being ‘completely as instructed’). This method of measuring adherence to home-based exercise has previously been used in hip rehabilitation randomised controlled studies [5, 29].

The secondary outcome measures and instruments will be:

- ***EuroQuol-5D(EQ-5D-3L)***. The EQ-5D is a standardised, validated questionnaire for the self-rating of a patient’s health status [62]. It comprises a visual analogue scale (VAS) measuring self-rated health and a health status instrument consisting of a three-level response (no problems, some problems and extreme problems) for five domains [63]; (a) mobility, (b) self-care, (c) usual activities, (d) pain and discomfort and (anxiety and depression). A respondents EQ-VAS gives self-rated health on a scale where the endpoints are labelled ‘best imaginable health state’ (100) and ‘worst imaginable health’ (0). The EQ-5D has been shown to provide more exact ratings of everyday activities and their disease-related restrictions compared with other measures of health related quality of life (QOL) [64]. The inherent consistency of the EQ-5D test has been evaluated in the literature and shown good responsiveness to change in patients with hip conditions [65], and has been used in similar populations with similar musculoskeletal complaints to our study population [66].
- ***Hospital Anxiety and Depression Scale (HADS)*** *.* This self-report questionnaire measures mood disorder and is validated for use as a screening tool in the general population [67]. It contains 14 items, seven relating to anxiety and seven relating to depression, which are scored separately. A score of 0–7 indicates no anxiety or depression, 8–10 is viewed as ‘borderline’ and 11–21 indicates the presence of anxiety or depression [36]. Previous research has demonstrated a link between hip OA and psychological wellbeing [68]. The HADS will be used to identify if anxiety or depression are confounders to treatment response and predictors of treatment outcome.
- ***6-Minute Walk Test***. Physical performance measures should be used in combination with self-report measures in the assessment of physical function [69]. The 6-minute walk test (6MWT) measures the distance an individual is able to walk over a total of 6 min on a hard, flat surface [70]. The goal is for the individual to walk as far as possible (measured by metres covered) in 6 min. The individual is allowed to self-pace and rest as needed as they traverse back and forth along a marked walkway. Timed walk tests have previously been used as a direct measure of physical performance in exercise-based osteoarthritis trials [36, 71], with positive inter-rater reliability [ICC 0.95 (CI:0.90, 0.98)] reported in one hip OA study [72].
- ***The Modified Star-Excursion Balance (Y-Balance) Test***. Impaired standing balance has been reported in people with hip pain compared with age matched healthy controls [73], and the exercise intervention in this study aims to correct poor balance and postural control. Dynamic balance and postural control will be measured using the modified star excursion balance test (SEBT), known as the Y-Balance test [74]. The test is performed with the participant standing on one leg and then reaches with the free limb as far as possible along three lines positioned in anterior, posteromedial, and posterolateral directions (Fig. 2). The Y-Balance Test was developed to standardise performance of the SEBT incorporating those directional movements with the greatest accuracy in identifying lower extremity dysfunction [75, 76]. The test has demonstrated good intra-rater reliability with reliability coefficients (ICC 2,1) ranging from 0.67 to 0.96 [62], and is currently used as a measure of postural control in patients undergoing UK military hip pain rehabilitation [54].

### Sample size

The primary endpoint will be change from baseline to 12-weeks in (a) the HAGOS ‘physical function in daily living’ subscale and (b) VAS numeric pain rating. Sample size calculation is based on clinically relevant changes over the study period (e.g., score changes between baseline and follow-up should be, on average, subjectively perceptible by the participant). The minimum clinically important difference (MCID) in the HAGOS subscale has been suggested to be 4.8–5.2 points [53]. Between participant standard deviations have not been widely reported for the VAS using intra-articular hip pain patients [1, 10]. Therefore, the required sample using a MCID of 5.2 between groups on the HAGOS subscale and a standard deviation of 11 points, with a significance level of 0.05 (2 tailed) and a power of 80 %, is 43 per group. Allowing for an estimated drop out rate of approximately 15 % at the 12-week follow-up, a total of 100 participants will be recruited into the study. Based on current referral rates it is anticipated this sample target will be met from routine referrals to DMRC Headley Court and no additional strategies to promote enrolment are required. The sample size was calculated using G*Power software Version 3.0.10 (Franz Faul, Universitaet Kiel, Germany). There is currently no RCT reporting the effects of a residential MDT intervention versus outpatient individualised treatment approaching this sample size in the available literature.

### Statistical analysis

The statistical analysis will be undertaken in accordance with the intention-to-treat (ITT) principle. The ITT analysis will include all participants, including those who do not fully adhere to the protocol and those with missing outcome data. A per-protocol analysis will also be performed where appropriate. Demographic and clinical characteristics as well as baseline data will be presented to assess the baseline comparability of the two groups. Differences from baseline to each time point will be calculated for all primary and secondary outcomes. Descriptive statistics will be presented for each group as mean change, standard deviations and confidence intervals (CI) for continuous variables, and frequencies and percentages for categorical variables. A longitudinal repeated measures design will be used to assess changes from baseline, and to make between group comparisons for the continuous outcome measures. Between group comparisons will be made immediately post treatment (1 week residential group; 6 weeks outpatient group) and at 12 weeks.

The effect of the interventions over time will be compared using linear and mixed models. These models are chosen as they appropriately adjust for correlation that occurs from collecting multiple observations per participant [37]. At the 1,6 and 12 week time points, continuous variables will be analysed using a mixed model repeated-measure analysis of variance (ANOVA) model (with treatment as the between group factor and time as the repeated factor). The non-parametric equivalent or data transformation will be used if non-normal distributions apply. Where significant differences occur, post-hoc comparisons will be conducted using Tukey’s multiple comparison procedure. Differences in nominal/ordinal data will be analysed using the *χ* ^2^ test. A significance level of 0.05 will be set for any inferential statistics conducted. The inclusion of the post treatment (T2) measure may help reveal if any differences occur as a consequence of the different interventions (as T2 reflects the immediate post-treatment scores for each group). However, this is based on the assumption that any changes in outcome from T1 and T2 (over 1 and 6-weeks) are linear, and also between T2 and T3. We recognise if the trend is non-linear then we will have insufficient data points to model the outcome over time in a way that will enable us to compare the trajectory of patient outcomes using the 3 time points. If interim analysis reveals this to be the case, we will remove T2 measures from the final analysis.

Secondary analysis will be undertaken to assess the predictors of outcome at the primary endpoint (12 weeks post treatment commencement) in both groups, using multiple regression analysis. Predictor variables including age, gender, baseline pain, physical function, duration of symptoms, mood/anxiety, self-efficacy, co-morbidities and exercise adherence will be measured. Analyses will be performed separately for MDT and IP group treatments. Response variables will be change in HAGOS function in daily living score pre and post intervention (MCID > 5.2 points) and numeric pain rating on the VAS. All predictors will be checked for collinearity. Univariate logistic regression will be performed on all potential predictors and those associated with the outcomes will be entered into the multivariate logistic regression model. Variables with the lowest predictive value will be removed from the model if *p* > 0.05. Odds ratios and 95 % CI’s will be calculated for all final predictors.

No statistical adjustment will be made for multiple testing. All tests will be two-sided and carried out at the 5 % level of significance. Changes to the study design or analysis plan will be documented with full justification. A statistician will oversee the blinded analyses of the data. The statistical analysis procedure will be discussed, reviewed and co-worked with statisticians at the Department for Health University of Bath, and School of Clinical Sciences at Bristol University.

### Data storage and quality assurance

All information obtained in the conduct of this study will be treated as confidential and participant anonymity will be ensured throughout the study period. Electronically stored data will be identified by a password-protected participant ID code unique to the study. We will transcribe data from paper forms directly into a bespoke Military Hip Rehabilitation Outcome (MILO) study relational database (Concentrica Ltd, Cambridge, UK) based on server’s in the Academic Department of Military Rehabilitation, DMRC and the Department for Health, University of Bath. To minimise transcription error, we will use manual double data entry. RC, JB, AW and AB will have access to the final trial dataset.

### Adverse events

All researchers involved in the conduct and supervision of this study will receive extensive training in all aspects of the administration of the trial protocol. The training will comprise a 1-day programme delivered at DMRC Headley Court. All clinical and research staff will receive a brief detailing the procedures for identifying and reporting safety issues including the use of project adverse events forms. They will be informed of the role and responsibilities of the Independent Medical Officer (IMO) and the lead researcher who takes day-to-day responsibility for safety of the project. Reporting of safety incidents will be duplicated using existing DMRC clinical health and safety reporting procedures and in accordance with the principles of good clinical practice (GCP). In the unlikely event this study is prematurely terminated for any reason, the MoD research ethics committee will be informed and provided with the justification for this action.

### Publication policy

We will submit the results of our study for publication in a suitable journal regardless of the outcomes. The trial will be reported in accordance with the CONSORT statement [25]. The chief investigator will take responsibility for producing draft report manuscripts and all co-investigators will review and approve the study results prior to submission for publication. Authorship of all manuscripts and presentations will comply with the ICMJE “Uniform requirements for Manuscripts Submitted to biomedical Journals” [87].

### Timeline

Ethics approval was obtained from the UK Ministry of Defence research ethics committee in August 2015. Recruitment of a study administrator was undertaken in November 2015. Participant recruitment and data collection will commence in January 2016. All recruitment and data collection are expected to be completed by December 2017.

## Discussion

Hip arthroscopy and non-surgical interventions such as the treatments offered by physiotherapists, are recommended in the management of non-arthritic FAI [1, 9].

However, despite the popularity and acceptance of residential MDT rehabilitation in the UK military, there are no studies presenting empirical data on the structure, process or efficacy of non-arthritic hip pain care. Given the interest in this model across the wider healthcare sector, there is an immediate need to test the efficacy of residential rehabilitation practice in a randomised trial to ensure all future patients receive rehabilitation in the optimal clinical setting.

This paper presents the protocol for a randomised controlled trial that will compare the effects of a residential MDT intervention with conventional outpatient care on pain and physical function in young non-arthritic hip pain patients. The study will be the first RCT to evaluate the structure and process of residential versus outpatient treatment options in young active adults with intra-articular, non-arthritic hip pain. The study design includes methodological elements to minimise the potential for bias including a power calculation, randomisation and intention-to-treat analysis. The primary and secondary outcomes are reliable and valid self-report measures of function and pain, quality of life, and physical capacity tests including dynamic postural control, walking and HROM.

Bennell et al. [5, 28] have previously employed a semi-structured treatment programme similar to the out-patient intervention in our study. These authors highlight that whilst this approach restricts individual tailoring of the programme, it reduces treatment variation thereby ensuring the intervention is accurately reported and replicated. We chose not to include a ‘no treatment’ control arm as natural recovery is unlikely to occur in participants reporting functional impairments and pain at baseline [28], and imposing natural recovery could be considered unethical.

There are some potential limitations with the study design. It is not possible to blind the participants or supervising therapy staff in clinical trials requiring informed consent and we cannot discount the possibility of assessor bias. The lack of follow-up beyond 12-weeks will not capture any longer term benefits of rehabilitation and will restrict our analysis to the period of intervention and home-based programme. Additionally, the use of a well-defined military population undergoing residential rehabilitation may limit the generalisability of our results to other populations and settings. However, we believe our study will yield results of relevance to practitioners and young active adults (e.g., sports participants) undergoing rehabilitation for non-arthritic hip pain.

This study will use high quality methodology in accordance with the CONSORT statement for randomised controlled trials [25]. It will respond to the lack of randomised trials directly comparing a residential MDT intervention with usual out-patient care for non-arthritic hip pain. The results will add to the evidence-base and inform clinical practice for the management of intra-articular hip pain and FAI in young active adults.

## Additional file

Additional file 1: MILO Study Intervention Guide. (PDF 2127 kb)

## Abbreviations

ADMR: Academic Department of Military Rehabilitation
ANOVA: Analysis of variance
BMI: Body mass index
CI: Confidence intervals
CONSORT: Consolidation of standards of reporting trials
DMRC: Defence Medical Rehabilitation Centre
ERI: Exercise rehabilitation instructor
FAI: Femoroacetabular impingement
GCP: Good clinical practice
HADS: Hospital anxiety depression scale
HAGOS: (Copenhagen) Hip and groin outcome score
HROM: Hip range of motion
ICMJE: International Committee of Medical Journal Editors
IMO: Independent medical officer
IP: Individual programme
ITT: Intention-to-treat
MCID: Minimal clinically important difference
MDT: Multidisciplinary team
MIAC: Multidisciplinary injury assessment clinic
MILO: Military hip rehabilitation outcome
MOD: Ministry of defence
MODREC: Ministry of Defence Research Ethics Committee
NAHS: Non-arthritic hip score
NSAID: Non-steroidal anti-inflammatory drugs
OA: Osteoarthritis
OARSI: Osteoarthritis Research Society International
OT: Occupational therapist
PCRF: Primary Care Rehabilitation Facility
PT: Physiotherapist
QOL: Quality of life
RCT: Randomised controlled trial
RRU: Regional Rehabilitation Unit
SEBT: Star excursion balance test
SIRBS: Sports injury rehabilitation beliefs survey
UK: United Kingdom
VAS: Visual analogue scale
