# Supplementary material for: A comparison of multidisciplinary team residential rehabilitation with conventional outpatient care for the treatment of non-arthritic intra-articular hip pain in UK Military personnel – a protocol for a randomised controlled trial
Source: BMC Musculoskelet Disord. 2016 Nov 8;17:459. doi: 10.1186/s12891-016-1309-z (PMC5100075; doi:10.1186/s12891-016-1309-z)
Supplement: Additional file 1: — MILO Study Intervention Guide. (PDF 2127 kb) [file 12891_2016_1309_MOESM1_ESM.pdf]

Defence Medical
Rehabilitation
Centre
Headley Court

# Military Hip Rehabilitation Outcome (MILO) Study

## Intervention Guide

**Chief Investigator Contact Details:**

Mr R Coppack
Academic Department of Military Rehabilitation
Defence Medical Rehabilitation Centre
Headley Court
EPSOM
Surrey
UK
KT18 6JW

Tel: 01372947103
E-mail: russ.coppack100@mod.uk

**Introduction**

This guide is intended to provide supervising clinicians and therapists with instruction, direction and guidance for the intervention employed in the Military Hip Rehabilitation Outcome (MILO) research trial. The intervention is developed following the Medical Research Council's framework for the development and evaluation of randomized controlled trials for complex interventions [1], and is based on the available published evidence and evidence-based research protocols for the conservative treatment of intra-articular hip pain, early hip osteoarthritis, and recommendations in clinical practice guidelines. [2-11]

The intervention is a semi-structured programme of exercise(s), manual therapy and education that can be individualised to each patient according to physical assessment findings and response to treatment. The guidance draws on the work of Bennell et.al [6,7] that recommends complex interventions used in research should be methodologically rigorous and reproducible, whilst reflecting "real-world" clinical practice. Therefore, whilst specific guidance is provided on exercise and treatment content, the supervising physiotherapist and therapeutic team can exercise professional judgement to determine how to integrate the protocol into the appropriate residential or outpatient treatment plan.

The goals of the programme are:

i. Restore / improve strength and neuromotor control of the deep hip stabilisers
ii. Restore / improve 'global' hip strength and neuromotor control
iii. Restore / improve core & trunk muscle function around the lumbar-pelvic-hip complex
iv. Address hip range of motion deficits
v. Maintain / improve aerobic fitness
vi. Patient education & advice

There is limited evidence and a lack of consensus on the most effective approach for the conservative management of intra-articular hip pain. Most rehabilitation programmes are reported in the scientific literature as clinical protocols only. This programme is therefore based on expert opinion from within and outside of UK defence rehabilitation, and an interpretation of the literature in this field. The programme will focus on modifying adverse hip joint forces through improvements in hip muscle strength and function, and modifying external joint loads[8]. The provision of appropriate education and advice is also crucial to the success of the programme.

All participants will be instructed to perform unsupervised home exercises 3-4 times/wk during the 3-month follow-up. Specific guidance on the choices and constraints for selection and dosage of home exercises is also provided.

| **Goal of Treatment - 1** | **Exercise** | **Description** | **Dosage** |
| --- | --- | --- | --- |
| **Restore & improve strength and neuromotor control of the deep hip stabilisers** | (1) Deep hip rotators lying face-down[1]<br><br>(2) Deep hip rotators in 4-point kneeling[1]<br><br>(3) Deep hip rotators in 4-point kneeling with hip external rotation (twisting foot inwards)[1]<br><br>(4) Deep hip rotators in 4-point-kneeling with hip internal rotation (twisting foot outwards)[1]<br><br>(5) Hip external rotation in 4-point kneeling with resistance band (Theraband ®)[1]<br><br>(6) Hip internal rotation in 4-point kneeling with resistance band (Theraband ®)[1]<br><br>(7) Hip external rotation with resistance (Theraband ®) and abduction with belt in 4-point kneeling[1]<br><br>(8) Hip internal rotation with resistance (Theraband ®) and abduction with belt in 4-point kneeling[1]<br><br>(9) Duck walk[1]<br><br>(10) Back Lying bridging through both limbs (stable surface). Progress through<br>- single limb (stable surface)<br>- both limbs (unstable surface / bosu ball)<br>- single limb (unstable surface / bosu ball) [2]<br><br>(11) Adductor magnus bridge progressions [2] | [1] Seven stage progressions described in the FAIR trial protocol (appendix 1, pages 17-18):<br><br>Bennell, K.L., O'Donnell, J.M., Takla, A., Spiers, L.N., Hunter, D.J., Staples, M., & Hinman, R.S. (2014). Efficacy of a physiotherapy rehabilitation program for individuals undergoing arthroscopic management of femoroacetabular impingement- the FAIR trial: a randomised controlled trial protocol. *Musculoskeletal Disorders, 15(58)*, 1-11.<br><br>[2] see DMRC adductor magnus bridge for exercise description and progressions (appendix 2, page 19-20 ).<br><br>---<br><br>- Commence activities in non-weight bearing positions and progress to weight-bearing and dynamic standing.<br><br>- Focus on strength and neuromotor control of the primary hip stabilisers. | [1] 1-minute 3-6 times per day individually tailored to patient needs based on physiotherapist assessment.<br><br>[2] - 3x8 repetitions<br>- 3x12 repetitions<br>- 3x20 repetitions<br><br>- dosage progression determined by the supervising physiotherapist. |

| Goal of Treatment - 2 | Exercise | Description | Dosage |
|---|---|---|---|
| **Restore & improve ‘global’ hip strength and neuromotor control** | (1) Quadriceps strengthening – progress through:<br><br>- sitting resistance (Theraband ®) band extension.<br>- partial squats.<br>- partial wall squats.<br>- sit-to-stand<br>- split squat to stand<br><br>(2) Arabesque[3]<br><br>(3) Squat with added (weight) resistance<br><br>(4) Single leg stance on unstable surface<br><br>(5) Single leg ‘shallow’ (45°) squat<br><br>(6) Sumo squat [4]<br><br>(7) Lunges<br><br>(8) Lunges with added resistance (hand held dumbbells)<br><br>(9) Side lunges<br><br>(10) Step ups / step downs<br><br>(11) Specific Gluteal and hamstring strengthening [5]<br><br>- Gluteal & hamstrings firing pattern<br>- Gluteal & hamstrings double leg bridge<br>- Gluteal & hamstrings single leg bridge<br>- Basic clam<br>- Clam hold<br>- Clam extension<br>- Leg rotation<br>- Leg rotation with circles<br>- Leg rotation with extension | - Ensure deep hip stabilisers have adequate strength and neuromotor control before undertaking global hip strengthening.<br><br>- Ensure deep hip stabilisers are active during progression of global hip strengthening.<br><br>- Include challenging functional neuromuscular balance / gait drills set – (see DMRC hip and groin course balance and proprioception programme – appendix 3, pages 21-22).<br><br>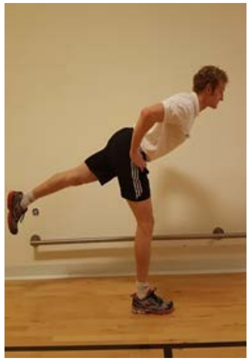 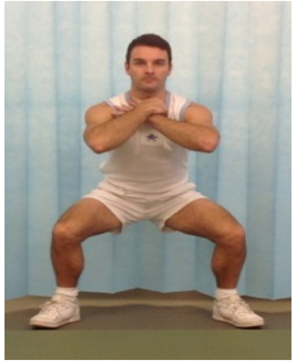<br><br>[3] Arabesque [4] Sumo Squat<br><br>[5] For exercise descriptions and progressions see DMRC Gluteal / Hamstrings programme (appendix 4, pages 23-25). | 3 x 8/10/12 repetitions; 3-4 times per week.<br><br>- exercise dosage, difficulty and progression determined by the supervising physiotherapist. |

| | | | |
|---|---|---|---|
| **‘global’ hip strength & neuromotor control (cont’)** | - Fig 4 clam<br>- Fig 4 clam hold<br>- Fig 4 clam hold<br>- Gluteus medius control (standing with ball) | [5] For exercise descriptions and progressions see DMRC Gluteal / Hamstrings programme (appendix 4, pages 23-25). | |

[3] **Kemp, J., & Crossley, K. (2014)**. Conservative management of femoroacetabular impingement: a case study and rationale for treatment. *ASPETAR sports medicine journal,3,*294-299.

| **Goal of Treatment - 3** | **Exercise** | **Description** | **Dosage** |
|---|---|---|---|
| **Restore & improve core & trunk muscle function around the lumbar-pelvic-hip complex** | (1) Double leg kneeling (plinth) [6]<br>(2) Pelvic dissociation (plinth) [6]<br>(3) Movement perturbation exercise [6]<br>(4) Trunk dissociation [6]<br>(5) Double leg kneeling (no feet support) [6]<br>(6) Single leg kneeling [6]<br>(7) Single leg dissociation [6]<br>(8) Single leg toe-taps [6]<br>(9) Double leg kneeling (airtex) [6]<br>(10) Pelvic dissociation (airtex) [6]<br>(11) Movement perturbation [6]<br>(12) Trunk dissociation [6]<br>(13) Double leg kneeling (no feet supt) [6]<br>(14) Single leg kneeling [6]<br>(15) Single leg dissociation [6]<br>(16) Single leg toe slides [6] | - Requires activation of transverse abdominis and the pelvic floor to maintain centralisation of the femoral head with lower extremity exercise.<br>- Ensure adequate control of the pelvis and spine is maintained during core activity.<br>- Ensure deep hip stabilisers are activated during core and trunk activity.<br>[6] For exercise descriptions and progressions see DMRC kneeling balance programme (appendix 5, pages 26-29). | [6,7] 1-minute 3-6 times per day individually tailored to patient needs based on physiotherapist assessment.<br>- dosage progression determined by the supervising physiotherapist. |

| | | | |
|---|---|---|---|
| **Restore & improve core and trunk muscle function around the lumbar-pelvic-hip complex (cont')** | (17) Back lying pelvic neutral [7]<br><br>(18) Pelvic neutral (4-point kneeling) [7]<br><br>(19) Salsa pelvic movements [7]<br><br>- lateral pelvic movements<br>- anterior/posterior pelvic movements<br>- hula hoop<br><br>(20) Bent knee fall out [7]<br><br>(21) Pelvic dissociation (standing) [7]<br><br>(22) Trunk dissociation (standing) [7]<br><br>(23) Trunk dissociation & balance (bosu ball) [7]<br><br>(24) Ball Squeeze and Bridge 0/30/45/60/90 degrees of flexion [8]<br><br>(25) Short Lever Adductor Side Bridge 1[8]<br><br>(26) Short Lever Adductor Side Bridge 2 [8]<br><br>(27) Long Lever Adductor Side Bridge [8]<br><br>(28) Adductor Magnus Bridge 1[8]<br><br>(29) Adductor Magnus Bridge 2 [8]<br><br>(30) Adductor Magnus Bridge Single Leg[8]<br><br>(31) Sumo Squat [8]<br><br>(32) Copenhagen adduction [8]<br><br>(33) Hip Abduction with Theraband [8]<br><br>(34) Sliding hip adduction/abduction [8] | [7] For exercise descriptions and progressions see DMRC hip dissociation programme (appendix 6, pages 30-33).<br><br>[8] For exercise descriptions and progressions see DMRC hip adductor loading programme (appendix 7, pages 34-36) | [6, 7] 1-minute 3-6 times per day individually tailored to patient needs based on physiotherapist assessment.<br><br>- dosage progression determined by the supervising physiotherapist. |

| Goal of Treatment - 4 | Exercise | Description | Dosage |
|---|---|---|---|
| **Address hip range of motion (ROM) deficits** | (1) Anterior hip stretch. [9]<br><br>(2) Rear hip (posterior capsule) stretch. [9]<br><br>(3) Hip flexion / extension in four point kneeling – “pendulum exercise”. [9]<br><br>(4) Muscle stretches (should match soft tissue / deep tissue massage techniques chosen from manual therapy options (see below).<br><br>- Hip extension<br>- Hip flexion<br>- Hip adduction and external rotation<br>- Hip internal rotation | Hip muscle stretching prescribed to stretch hip muscles and other surrounding soft tissue in order to increase hip ROM.<br><br>Manual therapy techniques are designed to improve the quality and ROM of the hip and surrounding soft tissue and to reduce pain. Distraction, passive joint mobilisation, sustained stretches and soft tissue techniques are recommended.<br><br>[9] For exercise descriptions and progressions see appendix 8 (page 37) adapted from the FAIR trial protocol.<br><br>Bennell, K.L., O’Donnell, J.M., Takla, A., Spiers, L.N., Hunter, D.J., Staples, M., & Hinman, R.S. (2014). Efficacy of a physiotherapy rehabilitation program for individuals undergoing arthroscopic management of femoroacetabular impingement- the FAIR trial: a randomised controlled trial protocol. *Musculoskeletal Disorders, 15(58)*, 1-11. | (1) 5-mins daily<br><br>(2) 3x30 seconds each session<br><br>(3) I-min daily<br><br>(4) 2-min total with 20 to 60s hold times. |
| | **Manual therapy techniques:** | | |
| | (a) Long-axis distraction with thrust. | (a) Supine hip is in 15°-30° of abduction, slight external rotation. Preferably use seat belt. Perform 3-6 thrusts at the beginning of the first set, then perform oscillations in the remaining sets. | (a) 3-6 sets of 30s |
| | (b) Seatbelt glide, or distraction mobilisations, with hip flexed. | (b) Supine with hip flexed and using a seatbelt, oscillatory passive accessory mobilisation forces applied caudally or laterally to the proximal thigh. | (b) 3-6 sets of 30s |
| | (c) Internal rotation in prone. | (c) Prone with knee flexed, internally rotate until contralateral pelvis rises, apply oscillatory force downward to contralateral pelvis. | (c) 3-6 sets of 30s |

| | | | |
|---|---|---|---|
| | (d) Soft tissue or deep tissue massage of quadriceps, adductors, hamstrings, psoas, lateral hip muscles, or posterior hip muscles and associated fascia. | (d) Firm effluage stroke, deep frictions or sustained pressure trigger point release with the muscle on stretch. | (d) 2-5 mins |
| | **Other optional techniques:** | | |
| | - Long-axis distraction in prone. | | 3-6 sets of 30s |
| | - Anteroposterior progression (posterior glide). | | |
| | - Posteroanterior progression (anterior glide). | | |
| | - Manual stretches to one joint knee extensor, rectus femoris, hip flexor, hamstring, hip internal rotator, hip external rotator or hip adductor. | | 6x20 secs or<br>4x30 secs or<br>2x60 secs |
| | Lumbar spine mobilisation | | 3-6 sets of 30s |

| **Goal of Treatment - 5** | **Exercise** | **Description** | **Dosage** |
|---|---|---|---|
| **Maintain / improve aerobic fitness** | (1) Stationary cycling. | (1) Upright bike with high seat to avoid excessive hip flexion past 90°. | Initially 15-20 mins at moderate intensity x 3 weekly. |
| | (2) Cross-trainer. | | |
| | (3) Swimming / walk in pool. | (3) Chest deep, forwards in a straight line initially; progress to pool running. | |
| | (4) Treadmill walk-run | (4) Forwards / backwards walk;jog;run.<br><br>The most appropriate mode of aerobic training should be prescribed by the supervising physiotherapist taking into account assessment findings and patient preferences. | |

| Goal of Treatment - 6 | Topic | Description | Dosage |
|---|---|---|---|
| **Patient Education** | (1) ‘About Hip Pain’ (PT)<br><br>(2) ‘Activity modification’ (OT)<br><br>(3) Benefits of exercise (ERI)<br><br>(4) Pain management (PN / OT) | - Members of the MDT to deliver education in a group setting to participants completing the residential treatment intervention.<br><br>- Physiotherapist to present information informally to each patient following the IP intervention at each outpatient appointment. | Arthritis Research UK (ARUK) patient information publications to be used as reference for education and advice.<br><br>All patients to be provided with copies of ARUK publications and pamphlets. |

PT = Physiotherapist; OT = Occupational Therapist; ERI = Exercise Rehabilitation Instructor; PN = Pain Nurse.

Appendix 9 - Additional Choices / Programmes for Supervising Physiotherapist to be selected based upon patient progression and individual needs (p.38-41)

**Programme Delivery and Progression Guidance[1]**

**Manual Therapy**

- The addition or removal of techniques is at the discretion of the physiotherapist based upon re-assessment and patient progress.

- During the early stages (session 1-2), a maximum of 2 techniques should be selected.

- If patients respond positively to treatment, and irritability is low, more techniques (including the optional techniques listed on page 7) can be introduced from session 3.

- Joint position can be altered or progressed in response to patient self-report and re-assessment findings.

- Accessory movements should (generally) be performed for 30 seconds. At least 3-sets should be performed, with a maximum of 6-sets if the patient is tolerating the technique and responding favourably. Physiological movements can be interspersed with sets of accessory movements.

**Home Exercise Progression**

- The home programme must include 4 to 6 exercises meeting the following general requirements:

  (a) To increase compliance, only 4 exercises should be used unless there is a strong clinical indication that the patient is adherent.

  (b) The exercises selected should be based upon the highest clinical priority established at the initial assessment. Exercise progression should be adjusted throughout the programme driven by changing priorities.

  (c) For strength-based exercises (treatment goals 1 and 2) the patient should be working at an intensity they rate between 5 to 8 on the 11-point Rating of Perceived Exertion (RPE) Scale. The resistance and exercise difficulty needs to be progressed to ensure strength gains are achieved.

  (d) For stretching exercises, the recommended dose is 2-minutes total for each stretch, comprising 2 or more repetitions with 20-to-60 second hold times.

  (e) For functional neuromuscular / neuromotor control drills the following principles should apply:

  - the most challenging drills / exercises the patient can complete safely should be selected.

  - the recommended dose is x2 exercises for 4 ‘practices’ x 30 seconds (4 minutes total).

  - drills can be changed each session to reflect re-assessment findings and changing treatment goals.

  - drills that re-enforce and complement improvements in strength and ROM should be applied in functional drills (i.e. hip abductor strengthening combined with standing dissociation or eccentric hip abductor control).

  (f) The residential and home exercise programme should include one of the aerobic training modes described in treatment goal 5. This should be completed 3-times per-week for 15-20 mins each session.

**Education and Advice**

- The education component is given in verbal and written forms and re-enforced throughout the period of the intervention. The physiotherapist should confirm the patients understanding of important concepts opportunistically at each session. The patient’s adherence to the home-exercise plan is discussed to identify individual barriers. Any obstacles to treatment implementation should be addressed by the physiotherapist and/or multi-disciplinary team.

[1] Progression guidance from a variety of published research protocols, clinical practice guidelines, published case-study, scientific literature and occupation specific expert opinion from within UK defence rehabilitation .

**Table 1 Overview of Outpatient Individual Programme (IP Group) and Residential (MDT Group) MILO Study Treatment Timetable**

| **Outpatient (IP) Protocol** | **Residential (MDT) Programme** |
|---|---|
| **Session 1 (45-60 mins)[†]**<br>• Subjective and objective assessment (20-25 mins)<br>• Patient education (5-mins)<br>• 1-2 manual therapy techniques (5-10 mins)<br>• Teach target exercise from treatment goals 1 and 2 (15-20 mins)<br>• Confirm home-exercises (5-minutes)<br>**Session 2 (45-60 mins)**<br>• Subjective and objective re-assessment (10-mins)<br>• Patient education (5-mins)<br>• Manual therapy techniques (15-20 mins)<br>• Teach revised home exercises; check log-book (15-20 mins)<br>**Sessions 3 to 8 (30-40 mins)**<br>• Subjective and objective re-assessment (5-mins)<br>• Manual therapy techniques (15-mins)<br>• Patient education & advice (5-mins)<br>• Progress home-exercises; check log-book; address adherence issues (15-mins) | **Day 1**<br>• Admission MDT clinic and baseline measures<br>**Day 2**<br>• Group-based introduction to treatment goal 1<br>• Group-based introduction to treatment goal 2<br>• Individual therapy appointments in accordance with patient timetable (PT/OT)[‡]<br>**Day 3**<br>• Group-based introduction to treatment goal 3<br>• Group-based introduction to treatment goal 4<br>• Group-based education topic 1 ('about hip pain')<br>• Individual therapy appointments in accordance with patient timetable (PT/OT)<br>**Day 4**<br>• Group-based introduction to treatment goal 5<br>• Consolidate individual patient exercise programme<br>• Group-based education topic 2 ('activity modification')<br>• Individual therapy appointments in accordance with patient timetable (PT/OT)<br>**Day 5**<br>• Group-based exercise targeting individual patient priorities<br>• Group-based education topic 3 ('benefits of exercise')<br>• Individual therapy appointments in accordance with patient timetable (PT/OT)<br>**Day 6**<br>• Group-based exercise targeting individual patient priorities<br>• Group-based education topic 4 ('pain management')<br>• Individual therapy appointments in accordance with patient timetable (PT/OT)<br>**Day 7**<br>• Confirm individual home-based exercise programme; issue log book;<br>• Discharge clinic with multi-disciplinary team |
| Follow-up period<br>• 4 to 6 home exercises, 3-times per week | Follow-up period<br>• 4 to 6 home exercises, 3-times per week |

[†] Timing does not include baseline outcome measures; [‡] PT = Physiotherapist; OT = Occupational Therapist.

**Table 2 - Treatment Techniques Session Planning and Home Exercise Selection Sheet**

| **Treatment Goal - 1** |
|---|
| **Restore & improve strength and neuromotor control of the deep hip stabilisers** |

| Exercise | Tick to select | Dosage (sets/reps/mins/sec) | Frequency (sessions per week) |
|---|---|---|---|
| Deep hip rotators lying face-down | | | |
| Deep hip rotators in 4-point kneeling | | | |
| Deep hip rotators in 4-point kneeling with hip external rotation (twisting foot inwards) | | | |
| Deep hip rotators in 4-point-kneeling with hip internal rotation (twisting foot outwards) | | | |
| Hip external rotation in 4-point kneeling with resistance band (Theraband ®) | | | |
| Hip internal rotation in 4-point kneeling with resistance band (Theraband ®) | | | |
| Hip external rotation with resistance (Theraband ®) and abduction with belt in 4-point kneeling | | | |
| Hip internal rotation with resistance (Theraband ®) and abduction with belt in 4-point kneeling | | | |
| Duck walk | | | |
| Back Lying bridging through both limbs (stable surface). Progress through<br>- single limb (stable surface)<br>- both limbs (unstable surface / bosu ball)<br>- single limb (unstable surface / bosu ball) | | | |
| Adductor magnus bridge progressions | | | |

| **Treatment Goal - 2** |
|---|
| **Restore & improve 'global' hip strength and neuromotor control** |

| Exercise | Tick to select | Dosage (sets/reps/mins/sec) | Frequency (sessions per week) |
|---|---|---|---|
| Quadriceps strengthening | | | |
| a. Partial squats | | | |
| b. Partial wall squats | | | |
| c. Sit-to-stand | | | |
| d. Split squat to stand | | | |
| e. Sitting resistance (Theraband ®) band extension. | | | |
| Arabesque | | | |
| Squat with added (weight) resistance | | | |
| Single leg stance on unstable surface | | | |

| |
|---|
| **Treatment Goal - 2 (cont')** |
| **Restore & improve 'global' hip strength and neuromotor control** |

| Exercise | Tick to select | Dosage (sets/reps/mins/sec) | Frequency (sessions per week) |
|---|---|---|---|
| Single leg 'shallow' (45°) squat | | | |
| Sumo squat | | | |
| Lunges | | | |
| Lunges with added resistance (hand held dumbbells) | | | |
| Side lunges | | | |
| Step ups / step downs | | | |
| Specific Gluteal and hamstring strengthening | | | |
| a. Gluteal & hamstrings firing pattern | | | |
| b. Gluteal & hamstrings double leg bridge | | | |
| c. Gluteal & hamstrings single leg bridge | | | |
| d. Basic clam | | | |
| e. Clam hold | | | |
| f. Clam extension | | | |
| g. Leg rotation | | | |
| h. Leg rotation with circles | | | |
| i. Leg rotation with extension | | | |
| j. Fig 4 clam | | | |
| k. Fig 4 clam hold | | | |
| l. Fig 4 clam hold | | | |
| m. Gluteus medius control (standing with ball) | | | |

| |
|---|
| **Treatment Goal - 3** |
| **Restore & improve core & trunk muscle function around the lumbar-pelvic-hip complex** |

| Exercise | Tick to select | Dosage (sets/reps/mins/sec) | Frequency (sessions per week) |
|---|---|---|---|
| Double leg kneeling (plinth) | | | |
| Pelvic dissociation (plinth) | | | |
| Movement perturbation exercise | | | |
| Trunk dissociation | | | |
| Double leg kneeling (no feet support) | | | |

| Treatment Goal - 3 (con't) |
|---|
| Restore & improve core & trunk muscle function around the lumbar-pelvic-hip complex |

| Exercise | Tick to select | Dosage (sets/reps/mins/sec) | Frequency (sessions per week) |
|---|---|---|---|
| Single leg kneeling | | | |
| Single leg dissociation | | | |
| Single leg toe-taps | | | |
| Double leg kneeling (airtex) | | | |
| Pelvic dissociation (airtex) | | | |
| Movement perturbation | | | |
| Trunk dissociation | | | |
| Double leg kneeling (no feet support) | | | |
| Single leg kneeling | | | |
| Single leg dissociation | | | |
| Single leg toe slides | | | |
| Back lying pelvic neutral | | | |
| Pelvic neutral (4-point kneeling) | | | |
| Salsa pelvic movements | | | |
| a. lateral pelvic movements | | | |
| b. anterior/posterior pelvic movements | | | |
| c. hula hoop | | | |
| Bent knee fall out | | | |
| Pelvic dissociation (standing) | | | |
| Trunk dissociation (standing) | | | |
| Trunk dissociation & balance (bosu ball) | | | |
| Ball Squeeze and Bridge 0/30/45/60/90 degrees of flexion | | | |
| Short Lever Adductor Side Bridge (1) | | | |
| Short Lever Adductor Side Bridge (2) | | | |
| Long Lever Adductor Side Bridge | | | |
| Adductor Magnus Bridge (1) | | | |
| Adductor Magnus Bridge (2) | | | |
| Adductor Magnus Bridge Single Leg | | | |
| Sumo Squat | | | |
| Copenhagen adduction | | | |

| **Treatment Goal - 3 (con't)** |
| --- |
| **Restore & improve core & trunk muscle function around the lumbar-pelvic-hip complex** |

| | | | |
| --- | --- | --- | --- |
| Hip Abduction with Theraband | | | |
| Sliding hip adduction/abduction | | | |

| **Treatment Goal - 4** |
| --- |
| **Address hip range of motion (ROM) deficits** |

| **Exercise** | **Tick to select** | **Dosage (sets/reps/mins/sec)** | **Frequency (sessions per week)** |
| --- | --- | --- | --- |
| Anterior hip stretch | | | |
| Rear hip (posterior capsule) stretch | | | |
| Hip flexion / extension in four point kneeling – "pendulum exercise | | | |
| Muscle stretches | | | |
| a. Hip extension | | | |
| b. Hip flexion | | | |
| c. Hip adduction and external rotation | | | |
| d. Hip internal rotation | | | |
| **Manual Therapy Techniques** | | | |
| a. Long-axis distraction with thrust | | | |
| b. Seatbelt glide, or distraction mobilisations, with hip flexed | | | |
| c. Internal rotation in prone | | | |
| d. Soft tissue or deep tissue massage of quadriceps, adductors, hamstrings, psoas, lateral hip muscles, or posterior hip muscles and associated fascia | | | |
| e. Long-axis distraction in prone | | | |
| f. Anteroposterior progression (posterior glide) | | | |
| g. Posteroanterior progression (anterior glide) | | | |
| h. Manual stretches to one joint knee extensor, rectus femoris, hip flexor, hamstring, hip internal rotator, hip external rotator or hip adductor | | | |
| i. Lumbar spine mobilisation | | | |

| **Treatment Goal - 5** |
|---|
| **Maintain / improve aerobic fitness** |

| Exercise | Tick to select | Dosage (sets/reps/mins/sec) | Frequency (sessions per week) |
|---|---|---|---|
| Stationary cycling | | | |
| Cross-trainer | | | |
| Swimming / walk in pool | | | |
| Treadmill walk-run | | | |

The most appropriate mode of aerobic training should be prescribed by the supervising physiotherapist taking into account assessment findings and patient preferences.

## References

[1] Campbell N.C., Murray, E., Darbyshire J., Emery, J., Farmer, A., Griffiths, F., Guthrie, B. et al,. (2007). Designing and evaluating complex interventions to improve health care: new guidance. *BMJ, 334,* 455-459.

[2] Wall, D.H., Fernandez, M., Griffin, R.D., & Foster, N.E. (2013). Nonoperative treatment for femoroacetabular impingement: a systematic review of the literature. *American Academy of Physical Medicine and Rehabilitation, 5*, 418-426.

[3] Hunt, D., Prather, H., Harris-Hayes, M., & Clohisy, J.C. (2012). Clinical outcomes analysis of conservative and surgical treatment of patients with clinical indications of prearthritic, intra-articular hip disorders. *American Academy of Physical Medicine and Rehabilitation, 4*, 479-487.

[4] Emara, K., Samir, W., Motasem, E.H., & Ghafer, K.A.E. (2011). Conservative treatment for mild femoroacetabular impingement. *Journal of Orthopaedic Surgery, 19(1),* 41-45.

[5] Wright, A.A., Hegedus, E.J., Taylor, J.B., Dischiavi, S.L., & Stubbs, A.J. (2016). Non-operative management of femoroacetabular impingement: a prospective, randomized controlled clinical trial pilot study. *J Sci Med Sport*, http://dx.doi.org/10.1016/j.jsams.2015.11.008

[6] Bennell, K.L., O'Donnell, J.M., Takla, A., Spiers, L.N., Hunter, D.J., Staples, M., & Hinman, R.S. (2014). Efficacy of a physiotherapy rehabilitation program for individuals undergoing arthroscopic management of femoroacetabular impingement-the FAIR trial: a randomised controlled trial protocol. *Musculoskeletal Disorders, 15(58)*, 1-11.

[7] Bennell, K.L., Egerton, T., Pua, Y.H., Abbott, H.J, Sims, K., & Buchbinder, R.(2011). Building the rationale and structure for a complex physical therapy intervention within the context of a clinical trial: A multimodal individualized treatment for patients with hip osteoarthritis. *Physical Therapy, 91(10),* 1525-1541.

[8] Kemp, J., & Crossley, K. (2014). Conservative management of femoroacetabular impingement: a case study and rationale for treatment. *ASPETAR sports medicine journal,3,*294-299.

[9] Eneski, K., Harris-Hayes, M., White, D.M., & Cibulka M.T. (2014). Non-arthritic hip pain: clinical practice guidelines linked to the international classification of functioning, disability, and health from the orthopaedic section of the American Physical Therapy Association. *J Orthop Sports Phys Ther*, *44(6),* A1-32.

[10] Tijssen, M., van Cingel. R.E.H., Staal, J.B., Teerenstra, S., de Visser, E., et al. (2016). Physical therapy aimed at self-management versus usual care physical therapy after hip arthroscopy for femoroacetabular impingement: study protocol for a randomized controlled trial. *Trials, 17(91),* doi 10.1186/s13063-016-1222-7

[11] Lewis, C.L., & Sahrmann, S.A. (2006). Acetabular labral tears. *Phys Ther*, *86,* 110-121.

**DMRC Headley Court**
**Centre for Lower Limb Rehabilitation**
**Hip & Groin**

## Deep Hip Stablisers - Appendix 1[b]

| | | |
|---|---|---|
| 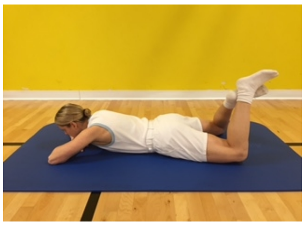 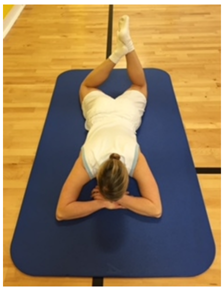 | **1. Deep hip rotators lying face-down**<br><br>Lie on your stomach with knees apart<br>Bend your knees so they are at 90 degrees; place the sole of your foot on your uninjured leg against the inner ankle on the other side. Gently press the ankle against the sole of the foot.<br><br>Keep your hamstrings and buttocks relaxed.<br><br>Hold for 3 seconds then relax for 2 seconds.<br>12 reps per minute.<br>1 minute | ☐<br>**Start date:**<br><br>**Date stopped:** |
| 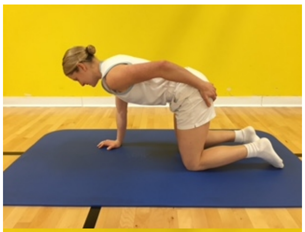<br>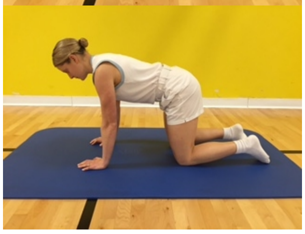 | **2. Deep hip rotators in 4-point kneeling.**<br><br>4pt kneeling, place your fingers on the bony part of your bottom called the ischial tuberosity. Move your fingers 2 cm out then 2cm up.<br><br>Imagine pulling your thigh up towards your pelvis, feel a gentle contraction of your QF muscle<br><br>Hold for 3 seconds then relax for 2 seconds.<br>12 reps per minute.<br>1 minute | ☐<br>**Start date:**<br><br>**Date stopped:** |
| 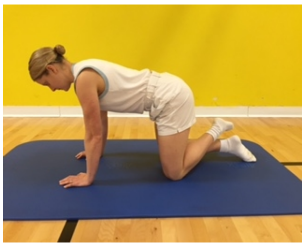 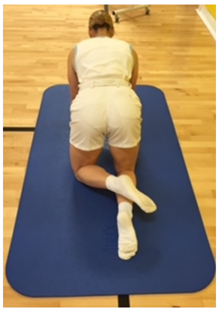 | **3. Deep hip rotators in 4-point kneeling with hip external rotation, (twisting foot inwards).**<br><br>4pt kneeling, activate QF muscle then gently rotate the foot on your injured leg inwards. Aim for your foot to be above the calf on your other leg. Slowly return to the starting position<br><br>Hold QF whilst you perform 5 reps, rest for 3 secs then repeat 5 again for minute | ☐<br>**Start date:**<br><br>**Date stopped:** |

[b] Appendix 1Adapted from Bennell, K.L., O'Donnell, J.M., Takla, A., Spiers, L.N., Hunter, D.J., Staples, M., & Hinman, R.S. (2014). Efficacy of a physiotherapy rehabilitation program for individuals undergoing arthroscopic management of femoroacetabular impingement-the FAIR trial: a randomised controlled trial protocol. *Musculoskeletal Disorders, 15(58)*, 1-11.

| | | |
|---|---|---|
| 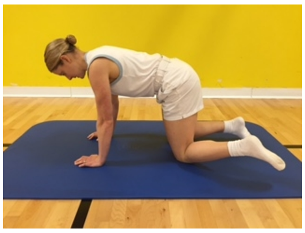 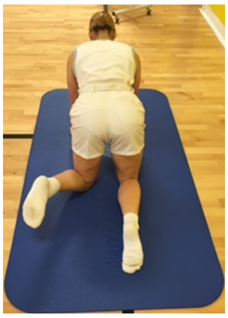 | **4. Deep hip rotators in 4-point kneeling with hip internal rotation, (twisting foot inwards).**<br><br>4pt kneeling, activate QF muscle then gently rotate the foot on your injured leg outwards. Aim for your foot to be above the calf on your other leg. Slowly return to the starting position<br><br>Hold QF whilst you perform 5 reps, rest for 3 secs then repeat 5 again for minute | ☐<br>**Start date:**<br><br>**Date stopped:** |
| 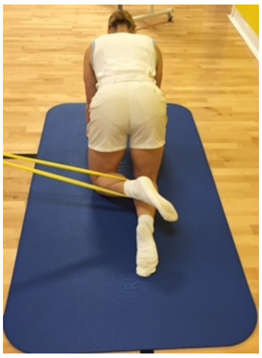 | **5. Hip external rotation in 4pt kneeling with resistance band.**<br><br>4pt kneeling, with resistance band around your ankle. Activate QF muscle then gently rotate the foot on your injured leg inwards. Aim for your foot to be above the calf on your other leg. Slowly return to the starting position.<br><br>Rest for 3 seconds then repeat for 1 minute.<br><br>**7. ADD ABDUCTION BELT** | ☐<br>**Start date:**<br><br>**Colour of band**<br><br>**Progressed to 7.**<br><br>**Date stopped:** |
| 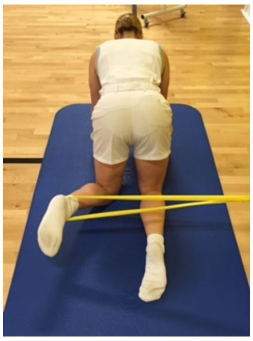 | **6. Hip external rotation in 4pt kneeling with resistance band.**<br><br>4pt kneeling, with resistance band around your ankle. Activate QF muscle then gently rotate the foot on your injured leg outwards. Aim for your foot to be above the calf on your other leg. Slowly return to the starting position<br><br>**7. ADD ABDUCTION BELT** | ☐<br>**Start date:**<br><br>**Colour of band**<br><br>**Progressed to 8.**<br><br>**Date stopped:** |
| | **9. Duck Walk** | ☐<br>**Start date:**<br><br>**Date stopped:** |
| 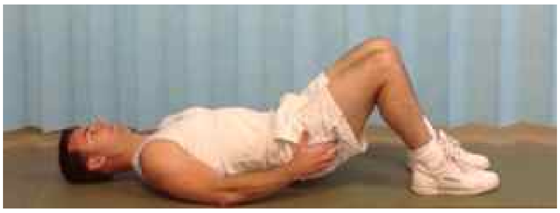 | **10. Bridging, Back Lying, both legs.** | ☐<br>**Start date:**<br><br>**Date stopped:** |

**Appendix 2 - Adductor Magnus Progressions:**

| | | |
|---|---|---|
| 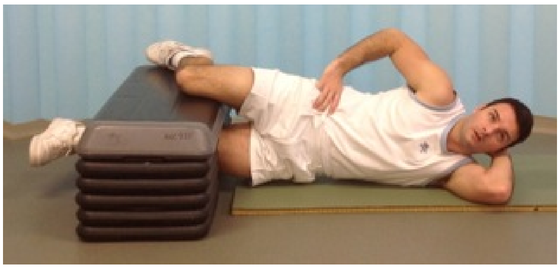<br>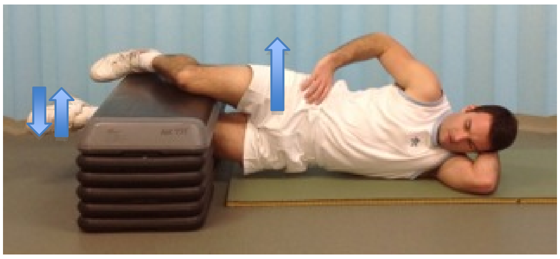<br>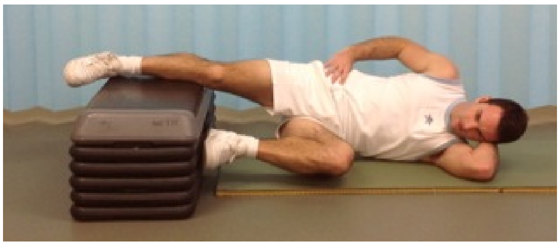<br>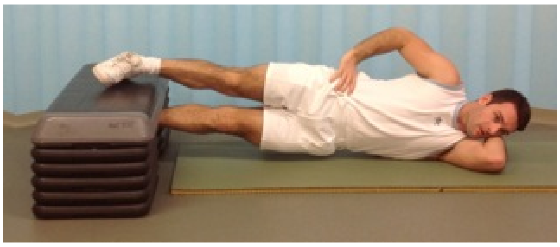 | Adductor Planks<br>65. Short Lever Static<br>Place the leg onto a bench or step box<br>Bend the knee to shorten the lever. Your shoulders, hips and knees should all be in a straight line<br>Both legs off the floor with the bottom leg out straight. Hold this position for 30 seconds and build progressively to 2 minutes.<br><br>66. Short Lever Adductor Dynamic<br>Starting position as above<br>Move the bottom leg up and down in small fast movements keeping the rest of your body stable<br>Perform small fast movements 30 times or for 30 seconds and build progressively to 2 minutes.<br><br>66 (a). Short Lever Adductor Dynamic Knee Tuck<br>As above moving the bottom leg towards your chest and back out to straight keeping trunk stable. Perform the movements 30 times or for 30 seconds.<br><br>67. Long Lever Static<br>Place the leg onto a bench/step box with the leg out straight<br>Your shoulders, hips and knees should all be in a straight line<br>Bottom leg of the floor<br>Hips move towards the floor and up again keeping the bottom leg off the floor.<br>Perform this exercise for 30 seconds progress up to 2 min. | ☐<br>**Start date:**<br>**a)**<br><br>**Date of Progress:**<br><br>**b)**<br><br>**c)**<br><br>**d)**<br><br>**Date stopped:** |
| 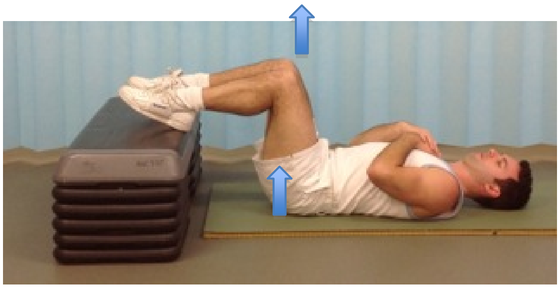 | Adductor Magnus Bridge<br><br>68. Activation<br>Place both your feet on the end of a bench/step box, the surface could move if you didn't control the exercise properly<br>Tuck your tail bone under and contract the hamstrings and adductors to move the hips off the floor a few centimetres.<br>12 repetitions x3 | ☐<br>**Start date:**<br>**a)**<br><br>**Date of Progress:**<br><br>**b)**<br><br>**c)**<br><br>**Date stopped:** |

| | | |
|---|---|---|
| 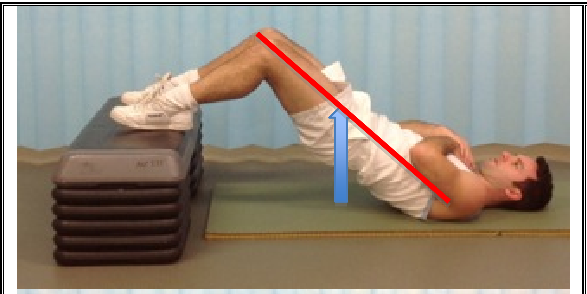 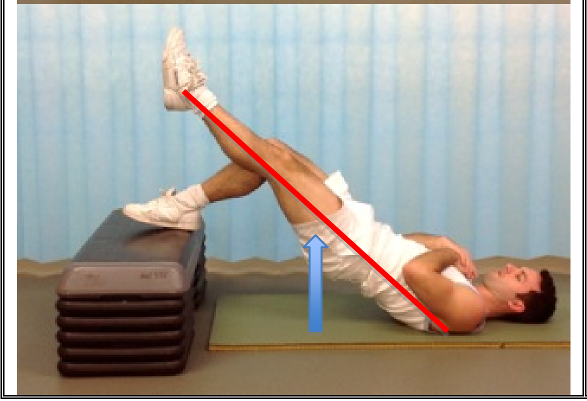 | 69.  Full Bridge<br>Start position and exercise as above.<br>Contract the hamstrings and adductors to move the hips up in line with the shoulders and knees.<br>Reps<br>20 repetitions x3<br><br>70. Single Leg<br>Start position as above<br>Hips up in line with your shoulder and knees<br>Once you are stable in this position extend one knew one your hips level.<br>8 repetitions x3 | |

**Appendix 3**

**DMRC Headley Court**

**Balance and Proprioception**

Balance refers to the ability to maintain bodily equilibrium, an equal distribution of weight e.g. enabling you to stand on one leg without falling over. Proprioception refers to the body's awareness of its position in space based on sensory information from joints, ligaments, muscles etc. For example, internal feedback mechanisms when you run will enable you to react to undulating surfaces without losing your balance.

Following pain or injury to your hip/pelvis /lumbar spine it is important to practice balance and proprioception exercises targeting the local area as well as the lower limb as a whole. Improving awareness of the body's position in space and ability to react to changes in external stimuli can help reduce the likelihood of further injury.

**What is the evidence?**

- Poor balance and proprioception is often observed following injury and is a good predictor of functional outcome
- Reduced standing balance has been demonstrated in people with low back pain
- Reduced ability to position the spine into a 'neutral' position has also been demonstrated in low back pain
- There is evidence to support that you are more at risk of re-injury if you do not rehabilitate your balance and proprioception
- Receptors are present in skin, muscles, joints, tendons, ligaments etc. and can be damaged when injured leading to altered sensory feedback

Pain and inflammation can also lead to alterations in sensory information and affect balance and proprioception.

## Standing Balance Exercises and Progressions

| | |
|---|---|
| 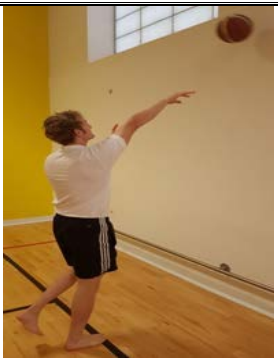 | **1. Single Leg Standing Throw**<br><br>Stand on your injured limb and throw a ball against the wall at a target and catch it again. Repeat 30 times.<br><br><u>Progression</u>:<br>a) Throw and catch with your dominant hand only.<br>b) Throw and catch with your non-dominant hand. |
| 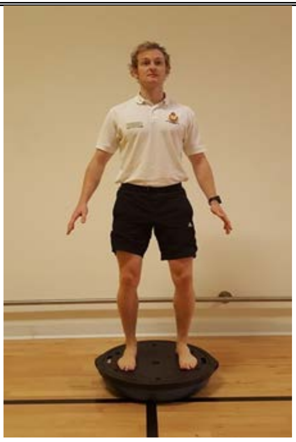 | **2. Bosu Ball Standing**<br><br>Stand on a bosu soft side up and ensure that your feet are spaced equally left and right and front and back.<br>Try to balance on the bosu and maintain your balance for as long as possible. Continue for 3 minutes.<br><br><u>Progression:</u><br>a) Place the arms across the chest.<br>b) Close your dominant eye.<br>c) Close both eyes.<br>d) With your eyes open, stand only on your injured limb.<br>e) All above with flat side up.<br><br>Note: Ensure the board is placed on a rubber mat and not directly on to the floor. |

| | |
|---|---|
| 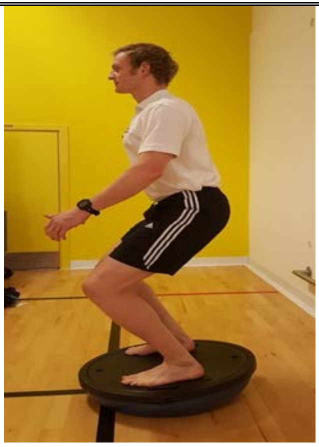 | **3. Bosu Ball Half Squats**<br>Stand on a bosu ball soft side up and ensure that your feet are spaced equally left and right and front and back and as wide as possible.<br>Try to balance on the bosu ball and then perform a slow and controlled half squat whilst maintaining good form.<br>Continue for 3 minutes.<br><br><u>Progression</u>:<br>a) Place the arms across the chest.<br>b) Close your dominant eye.<br>c) Close both eyes.<br>d) All above with the hard side up.<br><br>Note: Ensure the board is place on a rubber mat and not directly on to the floor. |
| 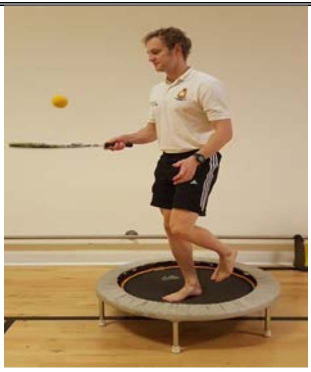<br>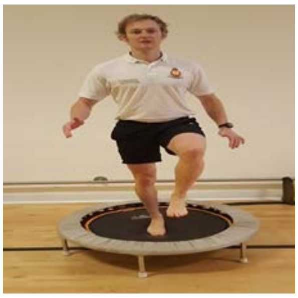 | **4. Trampette Exercises**<br><br>**a) Balance**<br>Whilst standing on your injured leg control the sponge ball up and down with the racket.<br>Continue for 3 minutes.<br><br><u>Progression</u>:<br>Place the trampette within a few feet of a flat wall and gently tap the ball against the wall.<br><br>**b) Jog and Stop**<br>Stand on a trampette and begin jogging on the spot. After 3-5 steps stop quickly and hold position for a couple of seconds then repeat.<br>Continue for 5 minutes. |
| 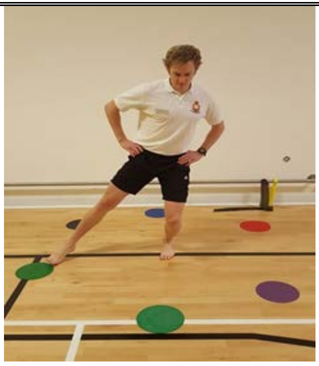 | **5. Clock Face Reaches**<br><br>Using Cones or Bean bags place around you to make a clock face. Progressively push each one out as far as you can reach without losing balance. Your foot is not to touch the floor on the reach out or return.<br>Continue for 5 min. |

**Appendix 4**

**DMRC Headley Court**

**Gluteals and Hamstrings**

**Muscle Activation – Gluteus Maximus/Hamstrings and Gluteus Medius**

**Why focus on activating Gluteus Maximus?**
Gluteus maximus is superficial muscle particularly important during weight-bearing functional activities which involves extending your leg (lower fibres of gluteus maximus) i.e. climbing stairs or abducting your leg i.e. controlling the pelvis when standing on one leg (upper fibres of gluteus maximus). A change in gluteus maximus activation is often seen in patients reporting hip pain, with a delay in the timing of activation and reduced activity during functional tasks. This in turn can lead to overloading of the hamstrings and shearing forces through the hip joint. Addressing these changes in muscle activation is an important part of your rehabilitation. Exercise for gluteus maximus should focus on the lower fibres of gluteus maximus that are most commonly affected and be initiated in weight-bearing aiming to reduce hip joint forces and ensure they are functional.

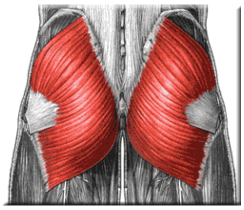

**What is the evidence?**
- Differences in the size of the lower fibers of gluteus maximus have been observed in people with mild osteoarthritis of the hip, with reduced size on the affected side.
- Individuals with advanced osteoarthritis of the hip, studies have found a significantly reduced size of upper fibres and lower fibres of gluteus maximus on the affected side, with more significant differences demonstrated in the lower fibres of gluteus maximus.
- Those with weak gluteus demonstrate higher anterior joint forces in hip extension. Gluteus maximus is maximally recruited during a lunge with the trunk flexed (weight-bearing and flexion).

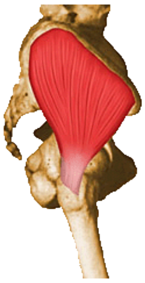

**Why focus on Gluteus Medius muscle activation?**
The lateral stabilizing system of the hips (gluteus medius) is particularly important when performing any single leg activities, such as walking and climbing stairs. People with hip dysfunction often demonstrate a change in pelvic-femur alignment during walking. This varies depending on stage of pathology and may alter the loading through the hip joint and increase the risk of further dysfunction

**What is the evidence?**
- Lack of deep gluteus medius could lead to increased superficial muscle activity and increased joint loading (**deep gluteus medius** most active in neutral abduction/adduction and **superficial gluteus medius** most active in 20 degrees adduction).
- MRI studies of people with advanced osteoarthritis hip have demonstrated reduced cross sectional area of gluteus medius (tensor fascia lata and upper gluteus maximus maintained size).
- In mild osteoarthritis hip there is an increased size of gluteus medius! This may be related to increased functional adduction and increased activation of superficial gluteus medius in a position of adduction.
- Careful rehabilitation is required as strengthening in adduction will lead to increased activity of the superficial system which could increase joint loading and compression of the iliotibial band on the tendon.

| | |
|---|---|
| 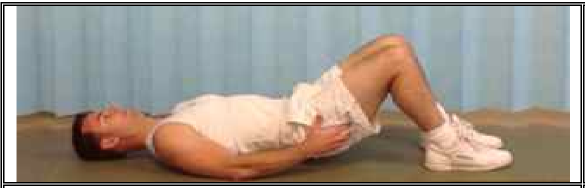 | **1 a. Gluts/Hamstrings Firing Pattern**<br>Place your thumb on your buttock and index finger on your hamstring (in the crease at the base of your bottom)<br>Contract your buttock and then your hamstring and initiate lifting your hips from the floor. |
| 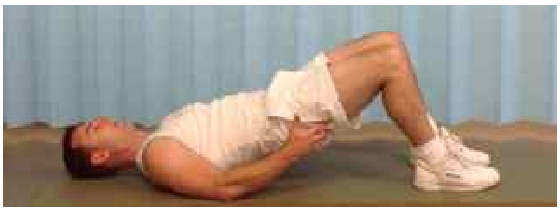 | **1b. Gluts/Hamstrings Double Leg Bridge**<br>Using the above firing pattern lift your buttocks up from the floor. Make sure you are slow and controlled with the movement up and down keeping the pelvis level at all times.<br><u>Progression</u>:<br>a) Arms out flat.<br>b) Elbows only<br>c) Arms across chest |
| 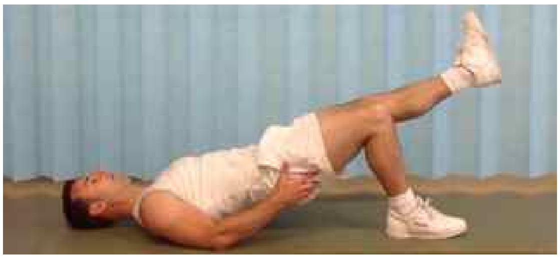 | **1 c. Gluts/Hamstrings Single Leg Bridge**<br>Same as above however when you achieve good stability at the top of range, extend one leg out straight whilst maintaining stability through the hip. Return the leg to the starting position before lowering the pelvis to the ground.<br><u>Progression</u>:<br>a) Arms out flat.<br>b) Elbows only<br>c) Arms across chest |
| 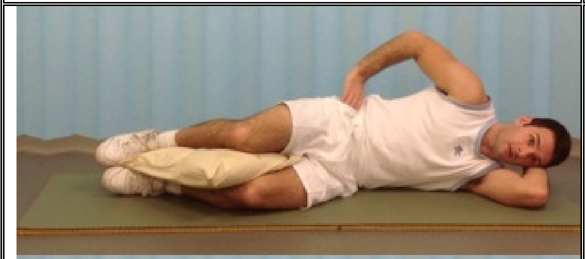<br>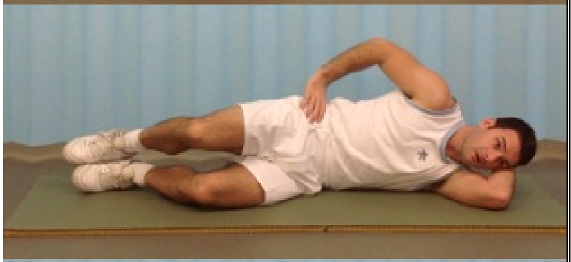<br>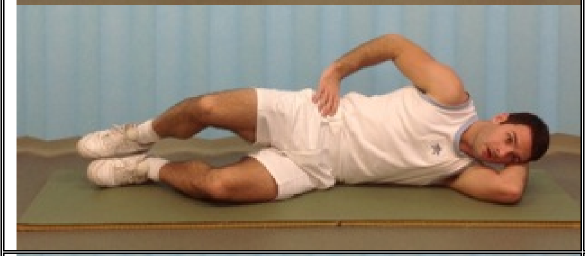 | **This exercise can be undertaken from the neutral position by placing a pillow between your knees or by actively holding the uppermost leg in a neutral position.**<br><br>**1. Basic Clam**<br><br>Lying on your side hips at 45 degrees<br>Straight line between heels, pelvis and shoulders<br>Knees at 90 degrees<br>Keep your feet together<br>Ensure the spine is set in spinal neutral position (straight line)<br>Slowly lift your top knee up and down under control isolating the movement from the hip.<br><br>**Don't allow the uppermost hip to 'roll out' and back.** |
| 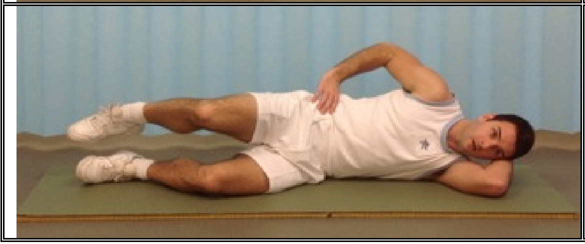 | **2. Clam Hold**<br><br>Position as above<br>Lift the knee and top foot 1 inch off the bottom foot, hold that position<br>Keep the knee higher than the foot |

| | |
|---|---|
| 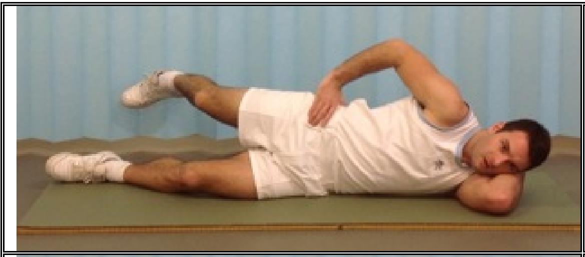 | **3. Clam Extension**<br><br>Same position as for 2<br>Slowly tap the top foot back behind the bottom foot onto the ground if you can<br>Foot relaxed<br>Bring the knee back to level then returned to the start position |
| 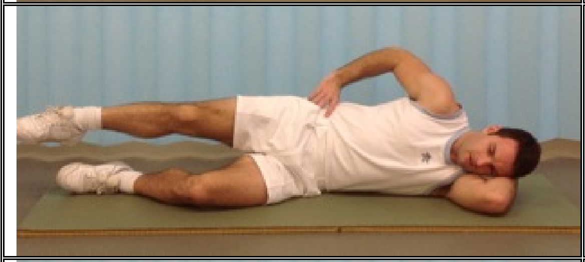 | **4. Leg Rotation**<br><br>Straighten the top leg in line with the body (knee, hip and shoulder in a straight line)<br>Keep the top leg straight, foot relaxed<br>Rotate the foot up towards the ceiling and back down from the hip. |
| 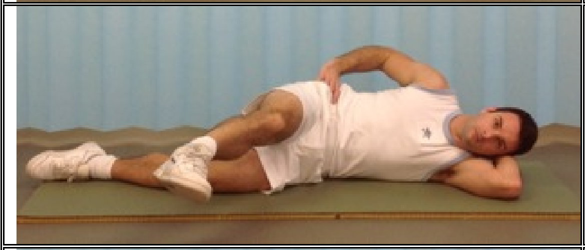 | **5. Leg Rotation with Circles**<br><br>Keep the leg straight, but bring it out in front of you to 90 degrees<br>Rotate externally from the hip<br>Perform small circles, rotating the whole leg away from the body. |
| 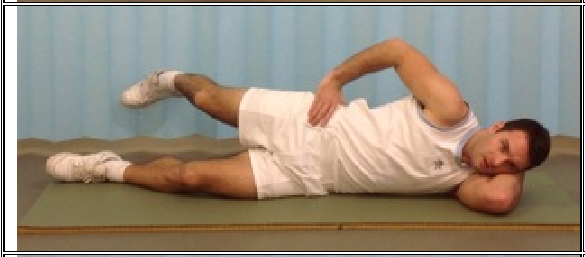 | **6. Leg Rotation with Extension**<br><br>Bring the leg back in line with the body<br>Rotate externally from the hip.<br>Take the leg backwards and away from your body<br>Aim to extend the leg 30-45 degrees from the body, but without using your back. |
| 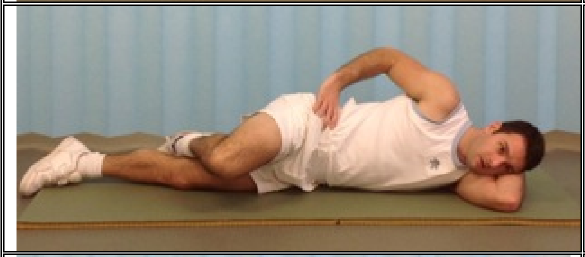 | **7. Fig 4 Clam Hold**<br><br>Position as above<br>Lift the top knee up and hold it there<br>Ensure your gluteuls are performing this exercise then feel the burn! |
| 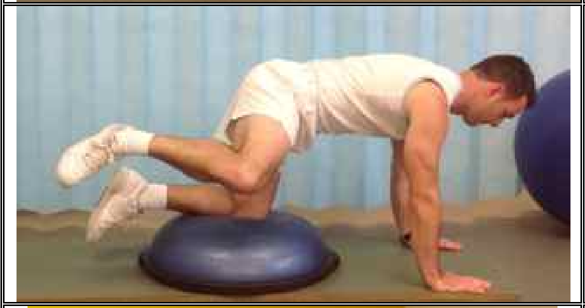 | **8. Glut Med Doggy Pee!**<br>Stabilize through your core<br>Rotate your leg out to the side and lift your knee up, start on a mat.<br><br><u>Progression</u>:<br>Do from a Bosu ball soft side up. |
| 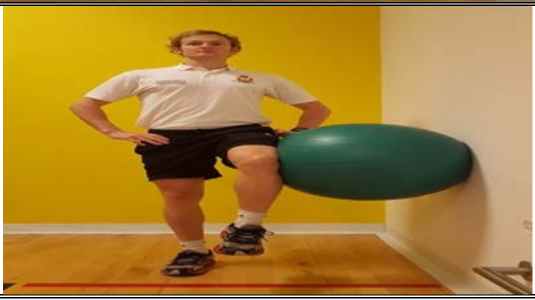 | **9. Glut Med Control**<br><br>Stand against a Swiss ball that is touching the wall<br>Apply a force through the side of your knee and into the ball<br>Lift your knee up to 90 degrees rolling the ball against the wall (this replaces clam shells) |

**Appendix 5**

**DMRC Headley Court**

**Kneeling Balance Programme**

| | |
|---|---|
| 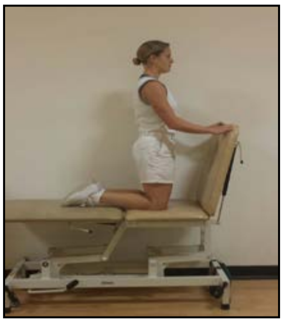 | **1. Double Leg Kneeling (Plinth)**<br>▪ Hold on to the top of the plinth.<br>▪ Keep a 90° angle at your knee.<br>▪ Keep hips in a straight line with your shoulders.<br><br>**Progression:**<br>a. Eyes closed.<br>b. No Hands<br>c. Turning head and shoulders. |
| 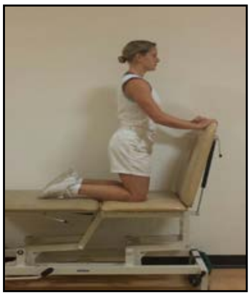 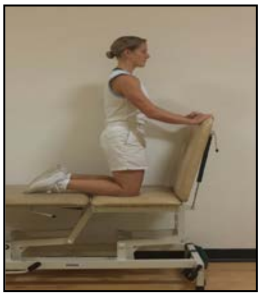 | **2. Pelvic Dissociation (Plinth)**<br>▪ Same starting position as above.<br>▪ Tilt your pelvis backwards and forwards – trying to tuck your tail bone under, then out again.<br>▪ Keep a 90° angle at your knee the whole time – you should not tip forwards or backwards.<br><br>**Progression:**<br>a. Eyes closed<br>b. No hands |
| 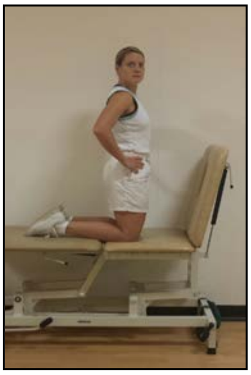 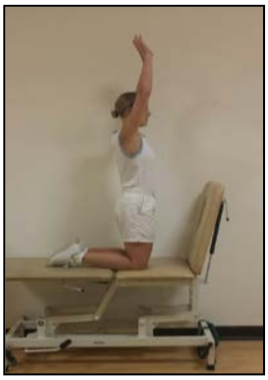 | **3. Movement Perturbation.**<br>▪ Same starting position as exercise 1, but place your hands on hips.<br>▪ Keep hips facing forwards and complete the below:<br><br>**Progressions:**<br>a. Turning head side to side.<br>b. Turning body side to side.<br>c. Shoulder press movement. |
| 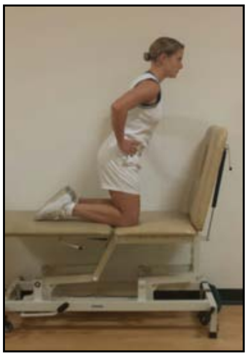 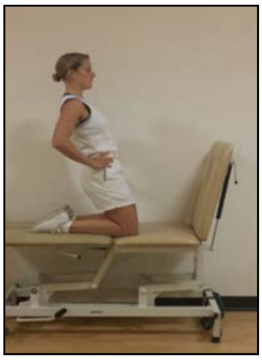 | **4. Trunk Dissociation**<br>▪ Same starting position as exercise 1, but place hands on hips.<br>▪ Keep your spine straight.<br>▪ Bend forwards from the hip.<br>▪ Return to the upright position.<br><br>**Progression:**<br>a. Same start position as above.<br>▪ Keep hips and spine in a straight line.<br>▪ Lean backwards from the knees.<br>▪ Return to the start position. |

| | |
|---|---|
| 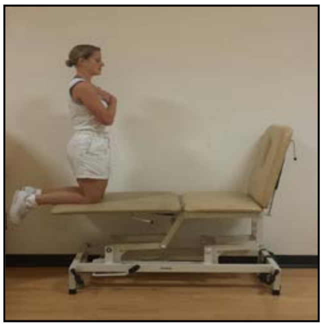 | **5. Double Leg Kneeling – No Feet Support.**<br>▪ Move to the end of the plinth.<br>▪ Your feet should hang over the edge.<br>▪ Arms folded across your chest.<br><br>**<u>Progression:</u>**<br>Complete all exercises in this new start position:<br>a. Ex's 1a-c<br>b. Ex's 2a-b<br>c. Ex's 3a-c<br>d. Ex's 4a |
| 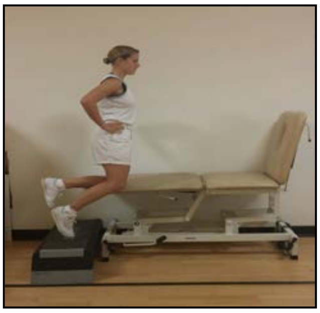 | **6. Single Leg Kneeling**<br>▪ Kneel with the leg you want to exercise on the plinth.<br>▪ Rest your other foot on a stool lower than the plinth.<br>▪ Raise your 'resting' foot up so that the bony parts of your pelvis are level.<br><br>**Hold this position for**:<br>a. 3x10 seconds.<br>b. 4x15 seconds.<br>c. 4x30 seconds. |
| 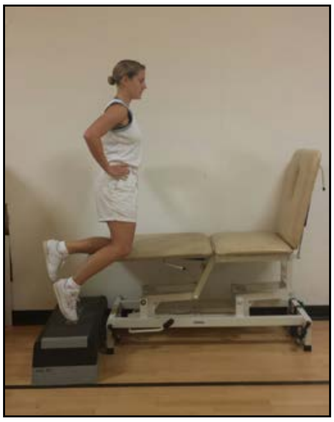 | **7. Single Leg Dissociation.**<br><br>▪ Start position same as for Exercise 6 (above).<br>▪ When you can maintain a level pelvis for 4x30 seconds you can move onto these exercises.<br><br>**Progression:**<br>a. Turning head side to side<br>b. Turning body side to side<br>c. Pelvic tilting. |
| 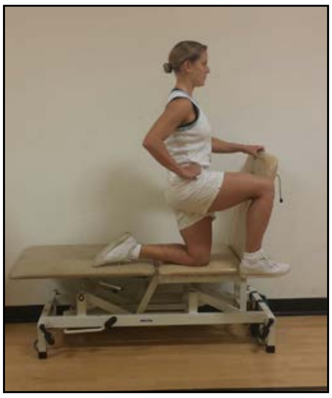 | **8. Single Leg Toe Taps**<br>▪ Kneel on a low plinth with the leg you want to exercise.<br>▪ Place your other foot lightly resting on the floor, stretched out to the side.<br>▪ Slowly take your foot off of the floor and flex your knee and hip to 90°.<br>▪ Pause for a couple of seconds.<br>▪ Slowly replace your foot to the floor behind you.<br>▪ Repeat |

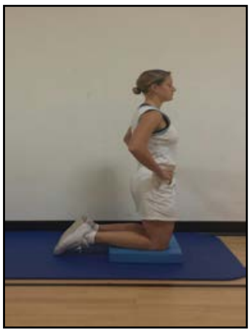

**9. Double Leg Kneeling (Airex)**
- Hands on hips. Toes resting on floor.
- Keep a 90° angle at your knee.
- Keep hips in a straight line with your shoulders.

**Progression:**
a. Eyes closed.
b. No Hands
c. Turning Head and shoulders.

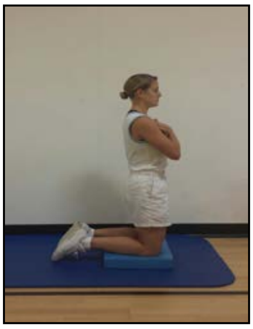 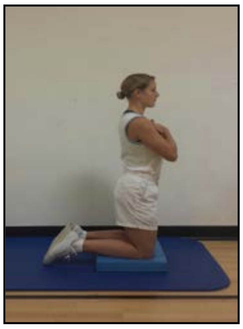

**10. Pelvic Dissociation (Airex)**
- Same starting position as exercise 1.
- Tilt your pelvis backwards and forwards – trying to tuck your tail bone under, then out again.
- Keep a 90° angle at your knee the whole time – you should not tip forwards or backwards.

**Progression:**
a. Eyes closed

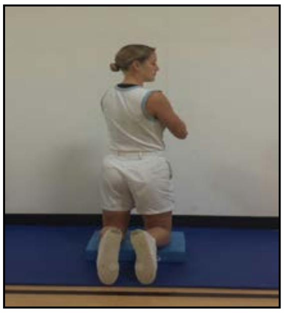

**11. Movement Perturbation.**
- Same starting position as exercise 1.
- Keep Hips facing forwards and complete the below:

**Progressions:**
a. Turning body side to side.
b. Shoulder press movement.
c. Throwing and catching ball against wall.

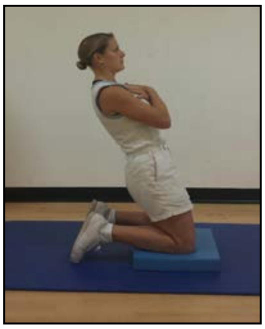 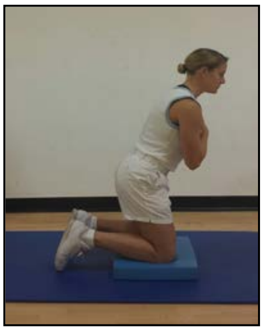

**12. Trunk Dissociation**
- Same starting position as exercise 1.
- Keep your spine straight.
- Bend forwards from the hip.
- Return to the upright position.

**Progression:**
a. Same start position as above.
- Keep hips and spine in a straight line.
- Lean backwards from the knees.

Return to the start position.

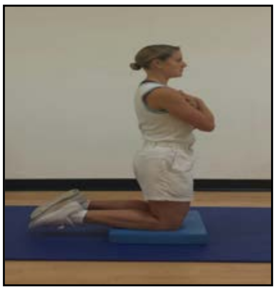

**13. Double Leg Kneeling – No Feet Support.**
- Make sure you are on a high enough step so that your toes cannot touch the ground.
- Arms folded across your chest**.**

**Progression:**
Complete all exercises in this new start position:
a. Ex's 1a-c
b. Ex's 2a
c. Ex's 3a-c
d. Ex's 4a

| | |
|---|---|
| 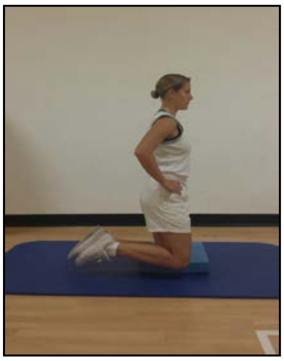 | **14. Single Leg Kneeling**<br>▪ Kneel with the leg you want to exercise on the airex cushion.<br>▪ Rest your other foot lightly on the floor.<br>▪ Raise your 'resting' foot up so that the bony parts of your pelvis are level.<br><br>**Hold this position for:**<br>a. 3x10 seconds.<br>b. 4x15 seconds.<br>c. 4x30 seconds. |
| 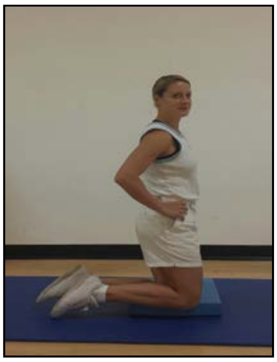 | **15. Single Leg Dissociation.**<br><br>▪ Start position same as for Exercise 6 (above).<br>▪ When you can maintain a level pelvis for 4x30 seconds you can move onto these exercises.<br><br>**Progression:**<br>a. Turning head side to side<br>b. Turning body side to side<br>c. Pelvic tilting. |
| 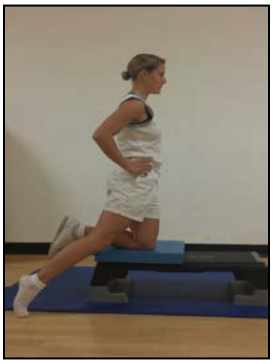 | **16. Single Leg Toe Slides.**<br>▪ Kneel on an airex cushion with the leg you want to exercise.<br>▪ Place your other foot lightly resting on the floor, stretched out to the side.<br>▪ Slowly slide your foot forwards.<br>▪ Pause for a couple of seconds.<br>▪ Slowly slide your foot to the side, pause.<br>▪ Slowly slide your foot behind you.<br>Repeat |

**Appendix 6**

**DMRC Headley Court**

**Hip Dissociation Programme**

**Why focus on dissociation exercises?**
Dissociation refers to the ability to isolate movement to a particular joint/area of the body. For example controlling your pelvis whilst lifting your thigh.

Difficulty with dissociation is frequently seen in people with back, pelvic and hip pain and can be related to changes in muscle activity/recruitment and reduced proprioception (see paragraph for balance and proprioception). Increased spinal stiffness, a protective mechanism in addition to reduced awareness of the position of the spine due to abnormal sensory information from the injured/painful area and will contribute to difficulties with dissociation. An example of this would be excessive spinal flexion associated with lifting the thigh.

It is important to note that there are many different variations in movement patterns observed between individuals with pain. Addressing the ability to dissociate is an important part of rehabilitation but must focus on **your** specific deficits as assessed by your therapist. You should initiate the exercises in a position where you are able to perform them efficiently; often lying on your back with knees bent is a good start. Once you are able to perform the exercise well you should you progress into functional positions, focusing on those you have difficulty with.

**What is the evidence?**

- Altered proprioception has been observed in many different conditions including back pain.
- Proprioception may be impaired secondary to altered sensory information received from the injured area as a result of pain, inflammation of injury to the sensory receptors directly
- Alterations in muscle activation have also been observed, frequently presenting with an increase in activity of the large superficial muscles. In the case of low back pain this will lead to an increase spinal stiffness and may reduce pain in the short-term, however in the long-term this will reduce the ability to dissociate and the ability to share the loading throughout the spine

| | |
|---|---|
| 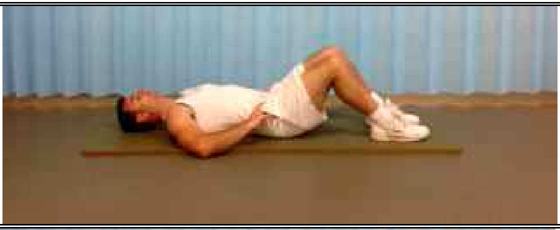 | **1. Pelvic Neutral**<br><br>In Crook Lying<br>Tilt your pelvis forwards and backwards |
| 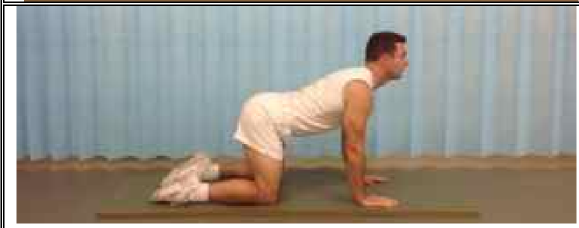<br>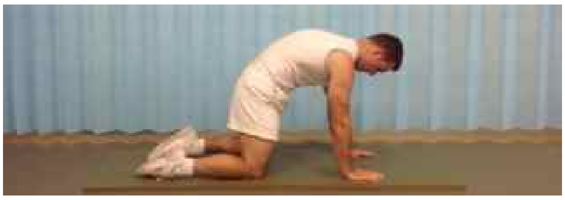 | **2. Pelvic Neutral**<br>**Cat/Cow**<br><br>Start with your knees below your hips and hands shoulder width apart.<br><br>Tilt your pelvis forwards and look upwards<br><br>Reverse the position to make a curve in your spine and tilt backwards putting your chin to your chest |

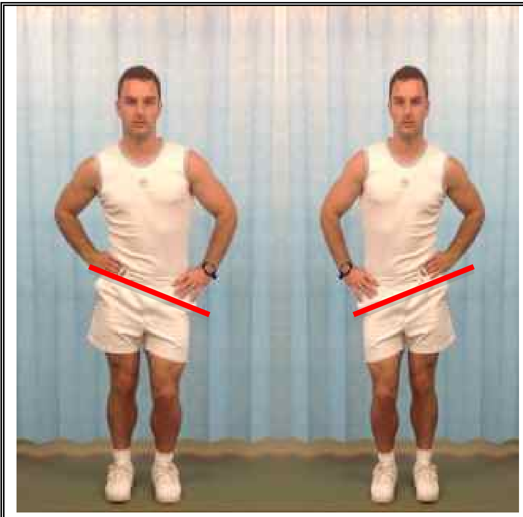

**3. Salsa moves**

**a) Lateral Pelvic Movements**

Stand in front of a mirror
Tilt your pelvis from side to side, you may use a little knee flexion (salsa dancing)
Use no trunk or shoulder movement

Progression:
i) kneeling on bosu ball
ii) kneeling on Swiss ball (with support in front)

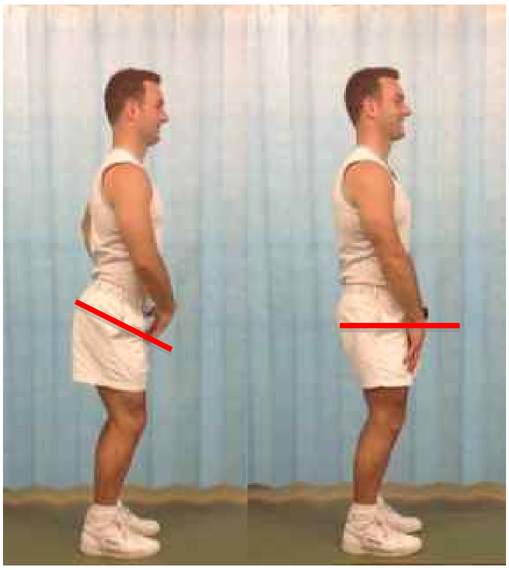

**b) Anterior/Posterior Pelvic movements**

Stand in front of a mirror
Tilt your pelvis forwards and backwards
Use no trunk or shoulder movement

Progression:
i) kneeling on bosu ball
ii) kneeling on Swiss ball (with support in front)

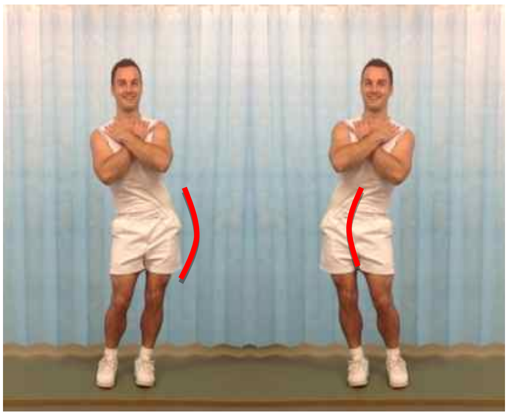

**c) Hula Hoop**

Stand in-front of a mirror
Put your hands across your chest
Rotate your pelvis in small circles in one direction and then reverse and change direction

Ensure that you use no trunk or shoulder movement

Progression:
i) kneeling on bosu ball
ii) kneeling on Swiss ball (with support in front)

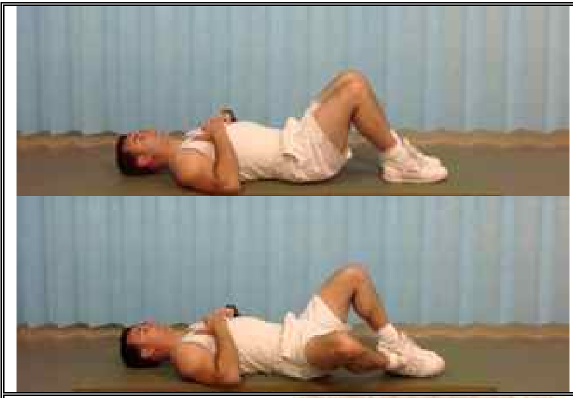

**4. Bent Knee Fall Out**

Keep your knee at 90 degs
Use a controlled movement to take your knee out to the side
Stop the movement when the pelvis starts to rotate and your opposite hip lifts off of the floor

<u>Progression</u>:
Do the same but with the hips and knees bent up to 90 degrees.

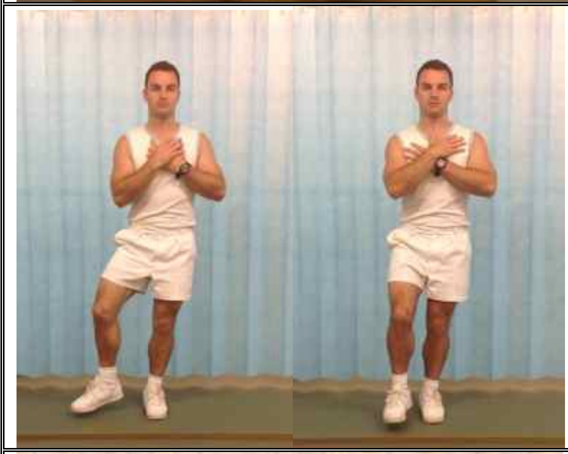

**6. Pelvic Dissociation**

Stand in front of a mirror
Start by lifting your knee to 30-45 degrees
Rotate your leg to the side without moving your trunk
Stop the movement when your pelvis starts to rotate with your leg

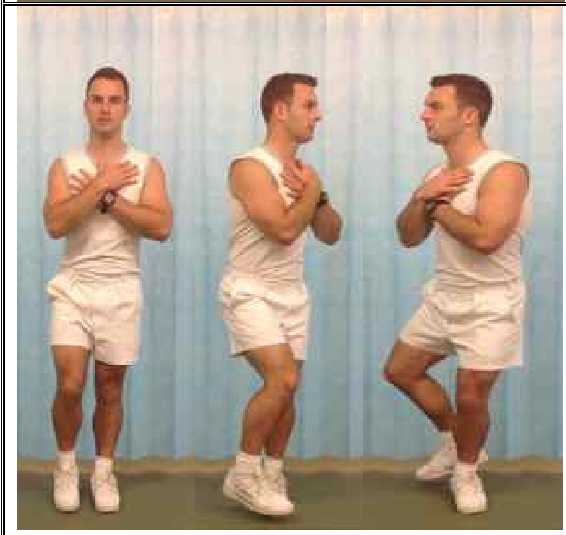

**7. Trunk Dissociation**

Stand on one leg and rotate your trunk and arms to the side, keep your supporting leg facing forwards

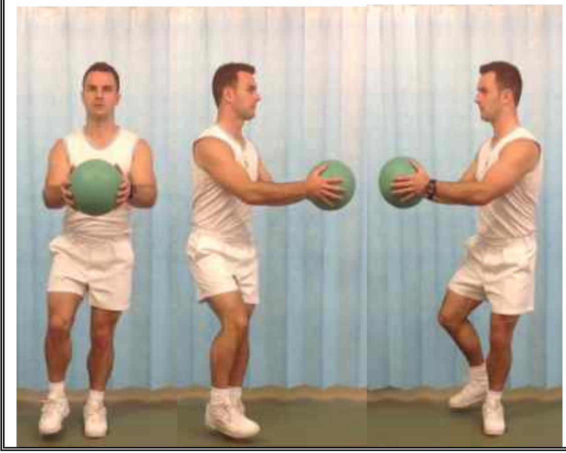

<u>Progression</u>:
a) Hold a medicine ball close to your chest and rotate from side to side
b) Repeat as above however extend your arms out straight so the ball is being held away from your body and rotate from side to side

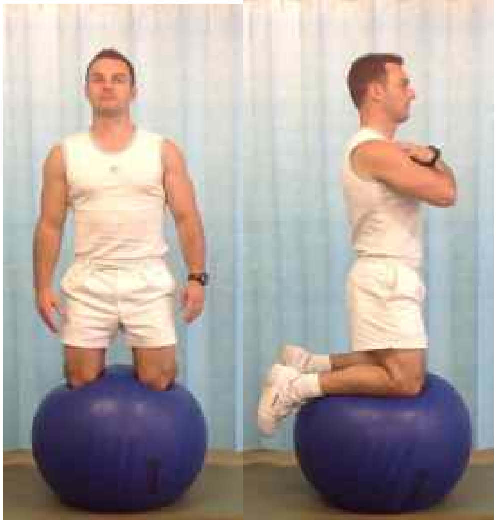

**8. Swiss Ball Balance**

**a) Anterior/Posterior pelvic tilt**
Kneel on a Swiss ball. If this is hard you can use support until you a comfortable to balance on your own
Tilt your pelvis forwards and back
Make sure you keep your trunk and shoulders still and the movement is only coming from your hips
Use a mirror to check your movements

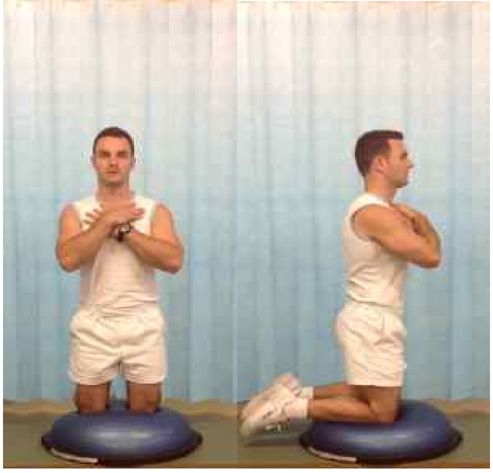

**9. Balance and Control**

Keel on a half ball/bosu ball initially get used to balancing without toes on the floor.
Progressions:

a) Weight transfer side to side.
b) Lift one knee to the side using a rotational movement from the hip

Note:
Keep your body upright and your pelvis in neutral.
Look forwards at a fixed point to help keep your balance.

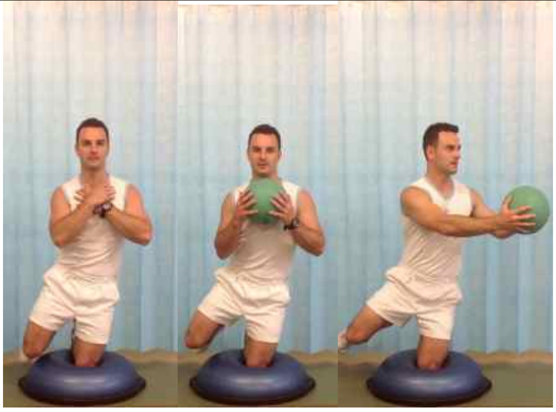

**10. Trunk dissociation and balance**

Kneel on a half ball/bosu ball
Lift one knee to the side using a abduction movement from the hip
Keep your body upright and your pelvis in neutral
Look forwards at a fixed point to help keep your balance

<u>Progression</u>:
a) Hold a medicine ball close to your body
Rotate from side to side holding the ball close to you trunk pivoting on the static hip/leg
b) Repeat as above however extend your arms out straight so the ball is being held away from your body and rotate from side to side.

**Appendix 7**

<table>
<tr>
<td>1.<br>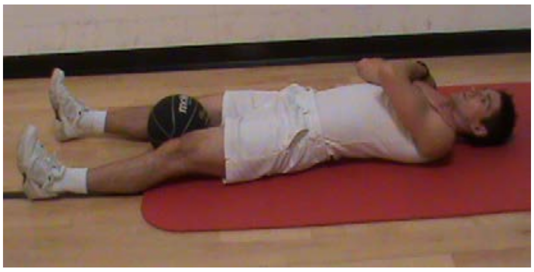<br>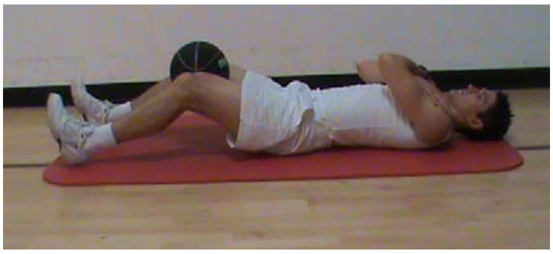<br>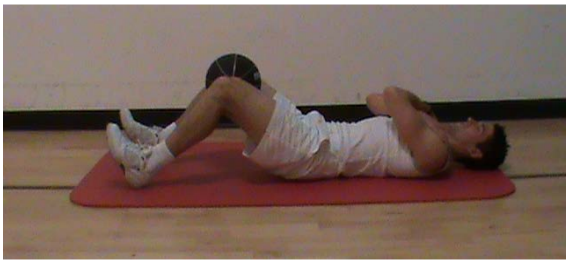<br>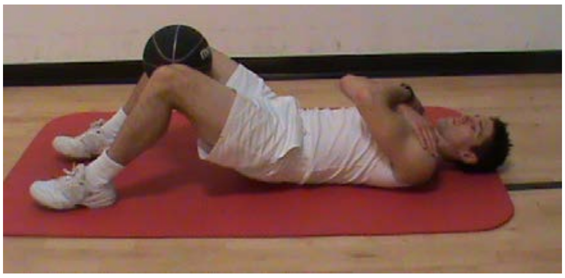<br>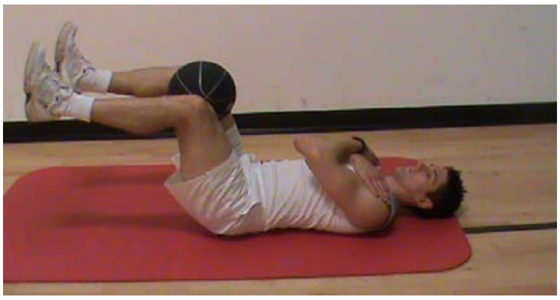</td>
<td>Ball Squeeze and Bridge<br><br>0/30/45/60/90 degrees of flexion<br><br>Squeeze and hold the ball for 6 seconds at 0/30/45/60/90 degrees to start with.<br><br>Reps<br>3 sets of 6 second holds<br><br>Progression<br>Build up towards 30 second holds.</td>
</tr>
</table>

| | |
|---|---|
| 2.<br>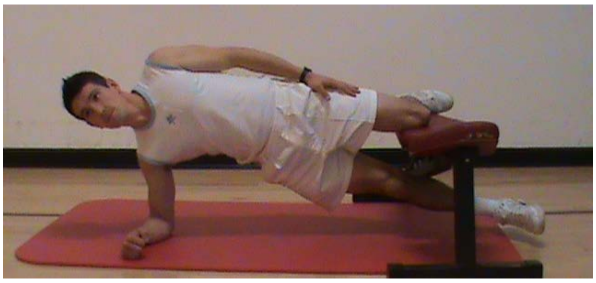 | **Short Lever Adductor Side Bridge**<br><br>Place the leg onto a bench bending the knee to shorten the lever. Your shoulders, hips and knees should all be in a straight line. Both legs off the floor with the bottom leg out straight. Hold this position for 30 seconds.<br><br>Reps<br>2 sets<br><br>Progression<br>Build up to 2 minute holds. |
| 3.<br>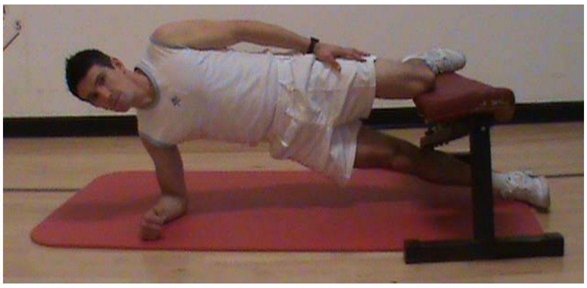 | **Short Lever Adductor Side Bridge**<br><br>Starting position as above then move the bottom leg up and down in small fast movements keeping the rest of your body stable. Perform the small fast movements 30 times or for 30 seconds.<br><br>Reps<br>2 sets<br><br>Progression<br>Build up to 2 minute holds |
| 4.<br>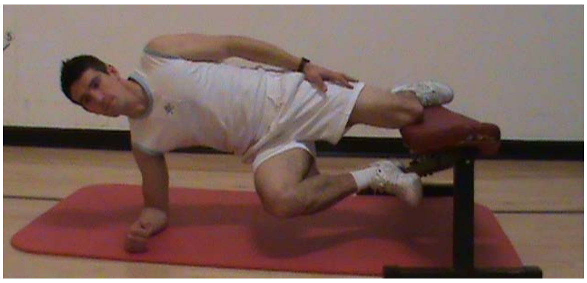 | **Short Lever Adductor Side Bridge**<br><br>As above moving the bottom leg towards your chest and back out to straight keeping trunk stable. Perform the movements 30 times or for 30 seconds.<br><br>Reps<br>2 sets<br><br>Progression<br>Build up to 2 minute holds |
| 5.<br>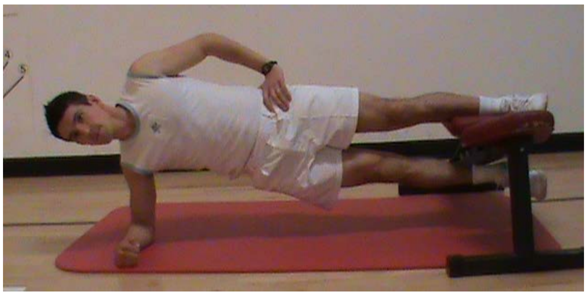 | **Long Lever Adductor Side Bridge**<br><br>Place the leg onto a bench with the leg out straight. Your shoulders, hips and knees should all be in a straight line. Both legs of the floor with the bottom leg out straight. Move the hips down towards the floor and up again keeping the bottom leg off the floor. Perform this exercise for 30 seconds.<br><br>Reps<br>2 sets |

| | |
|---|---|
| 6.<br>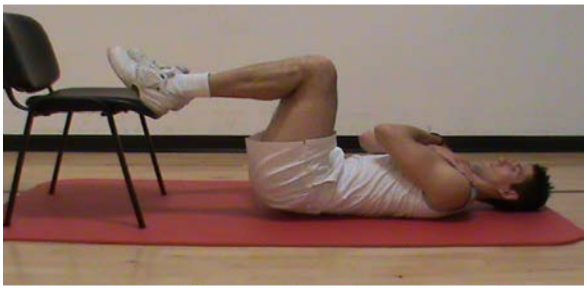 | **Adductor Magnus Bridge**<br><br>Place both your feet on the end of a bench or chair, the surface could move if you didn't control the exercise properly. Your hips should be bent to a 120 degree angle<br><br>Tuck your tail bone under and contract the hamstrings and adductors to move the hips off the floor a few centimetres.<br><br>Reps<br>12 repetitions of 3 sets |
| 7.<br>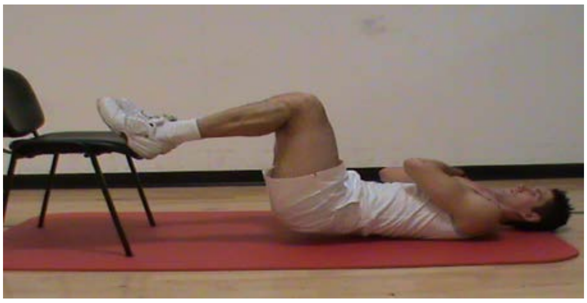 | **Adductor Magnus Bridge**<br><br>Start position as above but with the hips at a 90 degree angle.<br><br>Tuck your tail bone under and contract the hamstrings and adductors to move the hips up in line with the shoulders and knees.<br><br>Reps<br>20 repetitions x3 |
| 8.<br>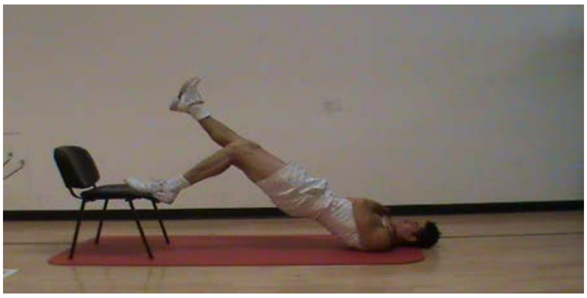 | **Adductor Magnus Bridge Single Leg**<br><br>Start position as above but bring your hips up in line with your shoulder and knees. Once you are stable in this position take away one leg keeping your hips level.<br><br>Reps<br>8 repetitions x3 |
| 9.<br>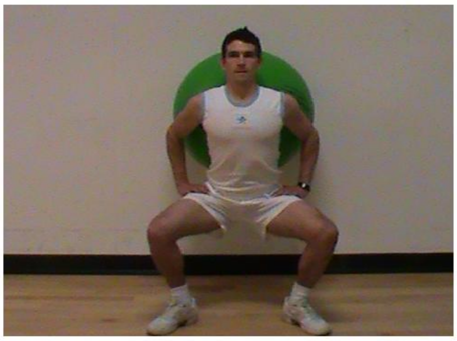 | **Sumo Squat**<br><br>Place the ball on your bottom. Turn your feet out 45 degree, put your weight on your heels and weight bear on the outside of your feet. Push your tail bone away from the ball drawing it under and up. Squat down slowly to past 90 degrees keeping your knees in line with your feet.<br><br>Reps<br>25 repetitions x3/4 every other day |

Appendix 8[c]

## Back of hip (posterior capsule) stretch

**Starting position**

Lie on your non-operated side.

Place a folded towel near the edge of the bed as shown in the picture.

**Exercise**

Bend your operated hip up as close to a right angle as is comfortable. Roll your hip forward and drop your knee over the edge of the bed.

**Dosage**

Take the stretch to the point of tension (not pain) and hold for 30 seconds. Do this three times.

**Indicators for progression**
Continue this exercise daily until the external rotation movement (dropping your knee out to the side when lying on your back) is equal to your non-operated hip

## Front of hip (anterior capsule) stretch

**Starting position**

Lying on your back on a bed. Bend up your non-operated leg.

Your bottom should be near the edge of the bed on your operated side.

**Exercise**

Take your operated leg out to the side and allow it to drop over the edge of the bed. Your thigh should still be supported by the bed.

You should feel a stretch in the front of your hip.

**Dosage**

5 minutes first thing in the morning, every day.

**Indicators for progression**
This exercise should be performed daily from two weeks after surgery until six weeks after surgery

[c] Appendix 8 Adapted from Bennell, K.L., O'Donnell, J.M., Takla, A., Spiers, L.N., Hunter, D.J., Staples, M., & Hinman, R.S. (2014). Efficacy of a physiotherapy rehabilitation program for individuals undergoing arthroscopic management of femoroacetabular impingement-the FAIR trial: a randomised controlled trial protocol. *Musculoskeletal Disorders, 15(58)*, 1-11.

**Appendix 9 - Additional Choices / Programmes for Supervising Physiotherapist to be selected based upon patient progression and individual needs.**

**Posterior Thigh Strengthening**

This is an overload programme. It is not about quantity but the quality of movement. Start with … sec per exercise and progress by 5 sec at a time until you reach 30 sec per exercise. You should feel a burning ache in your gluteal muscles by the end, if not you need to progress your timings. Try to keep working for around 4 minutes at a time.

| | | |
|---|---|---|
| 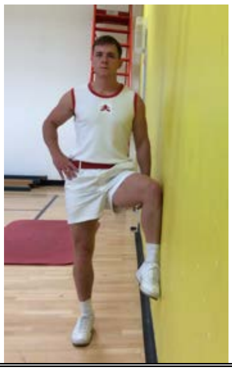 | **Wall Press:**<br>• In a single leg standing position with the non-weight bearing hip flexed to 90°.<br>• Rotate the flexed leg towards the wall keeping the pelvis facing forward and level.<br>• Press the inner leg firmly against the wall and hold for 20 secs.<br>• Return to starting position and repeat.<br><br>Gmed > Gmax | □<br>**Start date:**<br><br>**Date of Progress:**<br>**a.**<br>**b.**<br>**c.**<br><br>**Date stopped:** |
| 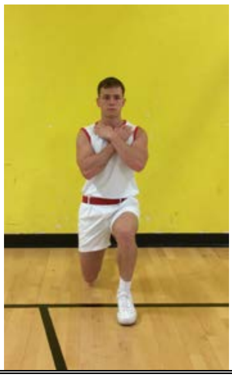 | **Lunge**<br>• In a standing position with the knees and feet hip width apart.<br>• Maintaining a neutral trunk position slowly lunge forward to a position of 90° bend of the front leg and return to the start position.<br>• Ensure you maintain good alignment of the leg and a level pelvis throughout the movement pattern.<br><br>Sets / Reps: | □<br>**Start date:**<br><br>**Date of Progress:**<br>**a.**<br><br>**Date stopped:** |
| 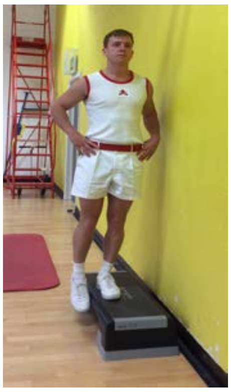 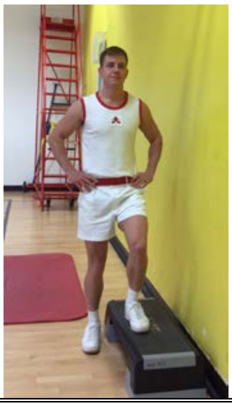 | **Lateral Step Up:**<br>• In a standing position with a step to one side and hand on the pelvis as demonstrated.<br>• Push up through the leg positioned on the step fully extending the knee and hip to complete the movement pattern.<br>• Return to start position and repeat.<br>• Ensure you maintain a level pelvis throughout the movement pattern.<br><br>Sets / Reps:<br><br>Gmax = Gmed | □<br>**Start date:**<br><br>**Date of Progress:**<br>**a.**<br>**b.**<br>**c.**<br>**d.**<br><br>**Date stopped:** |

| | | |
|---|---|---|
| 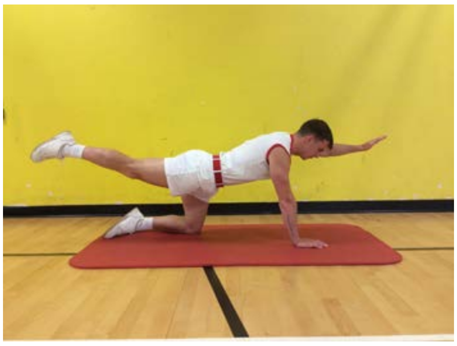 | **Point Kneeling Superman's:**<br><br>• In a 4 point kneeling position with a neutral lumbar position as demonstrated.<br>• Slowly extend one leg and opposite arm at the same time to the horizontal level position maintaining your back posture throughout the movement pattern.<br>• Return to the start position and repeat with the opposite arm / leg.<br><br>Sets / Reps:<br><br>Gmax > Gmed | □<br>**Start date:**<br><br>**Date of Progress:**<br>**a.**<br>**b.**<br>**c.**<br><br>**Date stopped:** |

**Standing Gluteal Activation Exercises**

| | | |
|---|---|---|
| 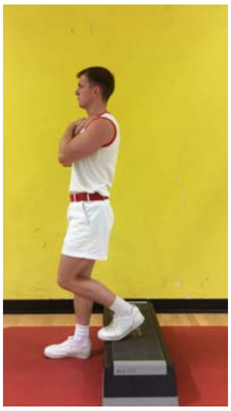 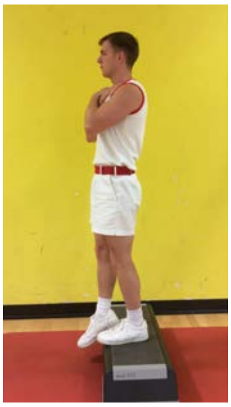 | **Retro Step Up:**<br><br>• Stand with a step set up as demonstrated.<br>• Place the leg performing the exercise backwards onto the step.<br>• Step up in a backwards motion onto the step fully extending the knee and hip to complete the movement.<br>• Return to start position and repeat.<br><br>Sets / Reps:<br><br>Gmax > Gmed | □<br>**Start date:**<br><br>**Date of Progress:**<br>**a.**<br>**b.**<br><br>**Date stopped:** |
| 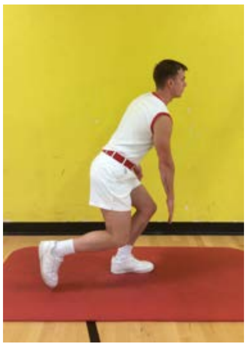 | **Single Leg Squat:**<br><br>• In a standing position on one leg.<br>• Slowly bend the hip and knee until you touch the mid point of the weight bearing foot with the opposite hand as demonstrated.<br>• Ensure you maintain a knee over foot position and neutral lumbar spine throughout the movement pattern.<br><br>Sets / Reps:<br><br>Gmed = Gmax | □<br>**Start date:**<br><br>**Date of Progress:**<br>**a.**<br>**b.**<br>**c.**<br><br>**Date stopped:** |

| | | |
|---|---|---|
| 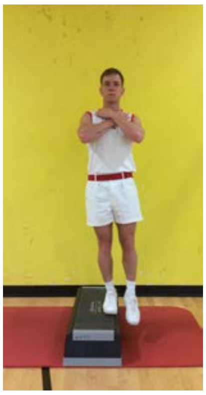 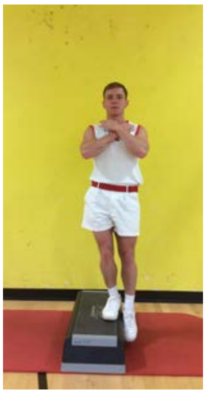 | **Pelvic Drop:**<br><br>• Using a step start in a sideways standing position on one leg.<br>• Maintaining a straight position of the weight bearing leg slowly drop the opposite side of the pelvis towards the floor.<br>• Draw the pelvis upwards back to a neutral hip level position.<br>Sets / Reps:<br><br>Gmed > Gmax | □<br>**Start date:**<br><br>**Date of Progress:**<br>**a.**<br><br>**Date stopped:** |
| 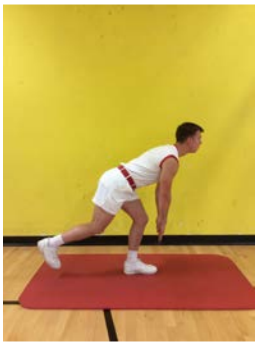 | **Single Leg Deadlift:**<br><br>• In a single leg standing position with the weight bearing leg flexed to approximately 30'.<br>• Slowly reach forward towards the floor bending the hip and trunk as demonstrated.<br>• Ensure you maintain a good knee over foot position throughout the movement pattern.<br>Sets / Reps:<br><br>Gmed = Gmax | □<br>**Start date:**<br><br>**Date of Progress:**<br>**a.**<br>**b.**<br>**c.**<br>**d.**<br><br>**Date stopped:** |
| 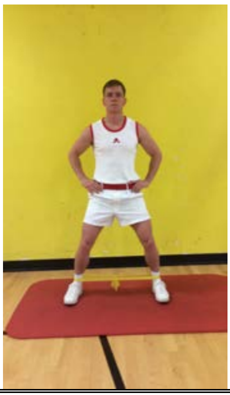 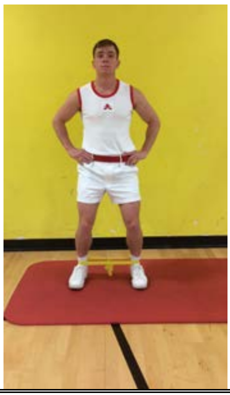 | **Lateral Band Walk:**<br><br>• In a wide stance with a resistance band positioned around the lower legs and hands on the pelvis as demonstrated.<br>• Step sideways against the resistance band in a slow and controlled fashion attempting to maintain a level pelvis throughout.<br>• Perform the required number of reps in one direction and repeat to opposite direction.<br>Sets / Reps:<br><br>Gmed > Gmax | □<br>**Start date:**<br><br>**Date of Progress:**<br>**a.**<br>**b.**<br>**c.**<br><br>**Date stopped:** |
| 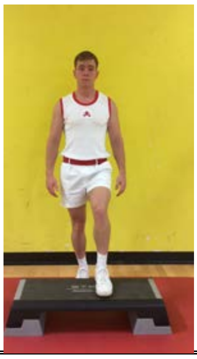 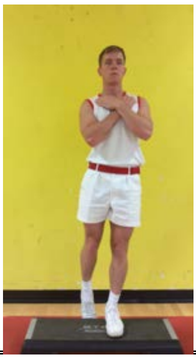 | **Forward Step Up**<br><br>• In a standing position with one leg placed onto the step.<br>• Lean forward and raise your body into a standing position on the step using the front leg.<br>• Fully extend the knee and hip to complete the movement.<br>• Ensure you maintain good alignment of the front leg throughout the movement pattern.<br><br>Gmax > Gmed | □<br>**Start date:**<br><br>**Date of Progress:**<br>**a.**<br>**b.**<br>**c.**<br><br>**Date stopped:** |

**Gluteus Medius Activation**

| | | |
|---|---|---|
| 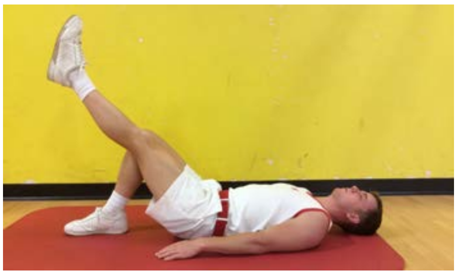<br>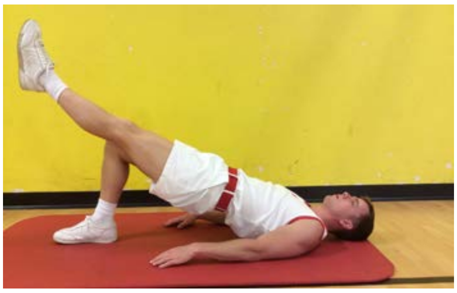 | **6. Unilateral Bridge:**<br><br>• In a supine position with both knees flexed to 90°.<br>• Extend one knee as demonstrated keeping the extended leg parallel to the opposite side.<br>• Lift the pelvis up to a bridge position using the weight bearing leg.<br>• Ensure that you maintain a level pelvis throughout.<br>• Return to starting position and repeat.<br><br>Sets / Reps: | □<br>**Start date:**<br><br>**Date of Progress:**<br>**a.**<br>**b.**<br>**c.**<br><br>**Date stopped:** |
| 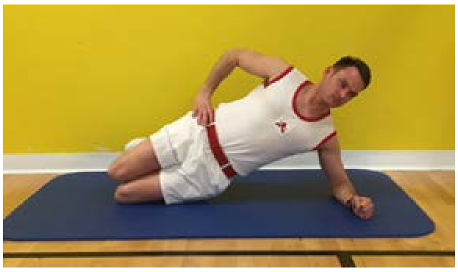<br>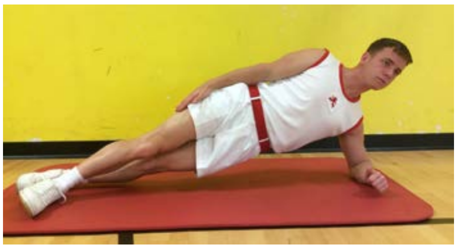<br>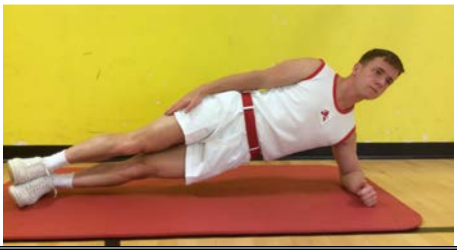 | **7. Side Bridge to Neutral Spine**<br><br>• Start from a side lying position with the knees bent, lower forearm resting on the floor<br>• Maintaining a neutral position of the lower back and hips slowly lift the hips to a position parallel with the floor.<br>• Ensure that the uppermost hip does not rotate during the exercise.<br>• Return to starting position and repeat.<br><br>Progression:<br><br>• Straighten legs - uppermost leg crossed over as demonstrated.<br>• Start from a side lying position with the uppermost leg resting on the lower leg.<br><br>Sets / Reps: | □<br>**Start date:**<br><br>**Date of Progress:**<br>**a.**<br>**b.**<br>**c.**<br><br>**Date stopped:** |

**Appendix 10 – Physical Examination and Outcome Assessment Test Procedures**

**A. Anterior Hip Impingement Test**

The patient is positioned in the back-lying position. The hip is passively flexed to 90˚ and then internally rotated and adducted as far as possible or to the first point of reported pain. The patient is asked what effect the motion has on symptoms. The test is considered positive if the patient reports the production of anterior hip pain[i]

**B. Resisted Straight Leg Raise**

The test is performed with the patient in back-lying with the hip in neutral and knee in full extension. The patient is asked to raise the leg while the examiner applies a counter-resistance to the anterior thigh just proximal to the knee. This test is felt to load the joint anterosuperiorly and reproduce anterior groin pain when an intra-articular lesion is present[ii]. We do not actively raise the leg to 30˚ before applying the external resistance as this can apply unwanted strain to the lumbosacral region[iii].

**C. The Thomas Test**

The patient is sat on the edge of the assessment couch with the examiner standing at the end of the couch directly facing the patient. The examiner passively lays the patient onto their back, bringing bi-lateral knees up to the patient's chest. The patient holds the non-tested leg toward their chest with bi-lateral arms as the examiner passively lowers the tested leg into extension. The examiner stabilisers the ipsilateral side of the pelvis with their other arm. A positive test is reproduction of a painful click or concordant groin pain.

**D. HROM – Internal Rotation**

The patient is positioned sitting with the hip at 90˚ of flexion with the lower-legs resting over the end of the assessment couch. The hip measured is placed in 0˚ of abduction, and the contralateral hip in approximately 30˚ of abduction to provide a firm base and comfort. The reference knee is flexed to 90˚, and the leg is passively moved to produce the desired hip rotation. The seated position assists to stabilise the pelvis and the pelvis is closely monitored to avoid unwanted pelvic motion. The motion is stopped when the examiner detects a firm end-feel or when pelvic movement is necessary for additional movement of the limb.

**E. HROM – Flexion**

The patient is positioned in the back-lying (supine) position with the hip in 0˚ of abduction, adduction and rotation. With the knee flexed, the hip is passively flexed while the lumbar spine is monitored to avoid posterior pelvic tilt. The motion is stopped when the clinician reaches a firm end-feel or when pelvic movement is necessary for additional movement of the limb. The examining physiotherapist will also assess joint play with particular attention to evidence of insufficient posterior glide[iv].

F. **HROM – Abduction**

The patient is positioned in back-lying with the hip in 0˚ of flexion and rotation. With the knee extended, the hip is passively abducted. Manual stabilisation is provided at the pelvis to prevent lateral pelvic tilt or pelvic rotation. The motion is stopped when the examiner reaches a firm end-feel or when pelvic movement is necessary for additional movement of the limb.

---

[i] Clohisy JC, Knaus ER, Hunt DM, Lesher JM, Harris-Hayes M, et al. Clinical presentation of patients with symptomatic anterior hip impingement. Clin Orthop relat Res, 2009; 467(3): 638-644.
[ii] Kelly BT, Williams RJ, Phillipon MJ. Hip arthroscopy: current indication, treatment options, and management issues. Am J Sports Med, 2003; 31: 1020-1037.
[iii] Martin RL, Eneski KR, Draovitch P, Trapuzzano T, Phillipon MJ. Acetabular labral tears of the hip: examination and diagnostic challenges. J Orthop Sports Phys Ther, 2006; 36(7):503-515.
[iv] Lewis CL, Sahrmann SA, Moran DW. Anterior hip joint force increases with hip extension, decreased gluteal force, or decreased iliopsoas force. Journal of biomechanics, 2007; 40(16): 3725-3731.

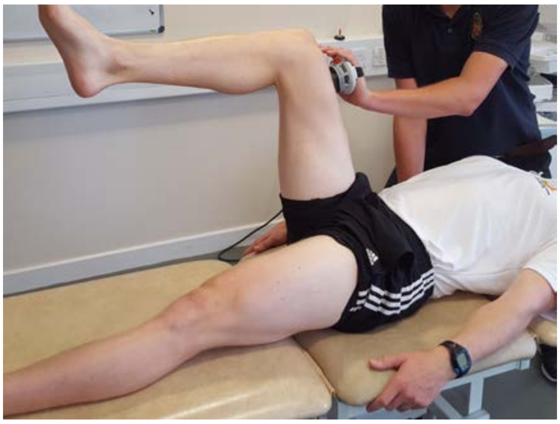

*(a)*

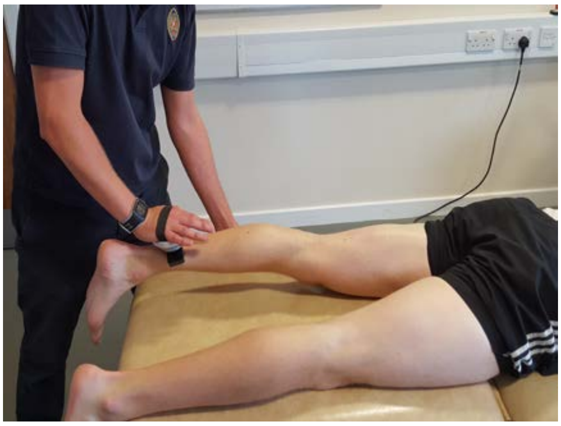

*(b)*

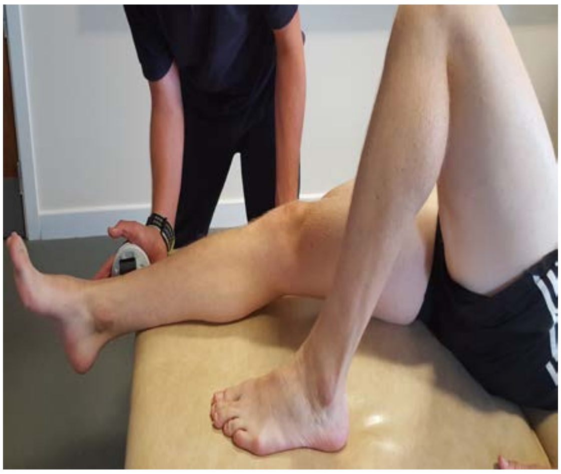

*(c)*

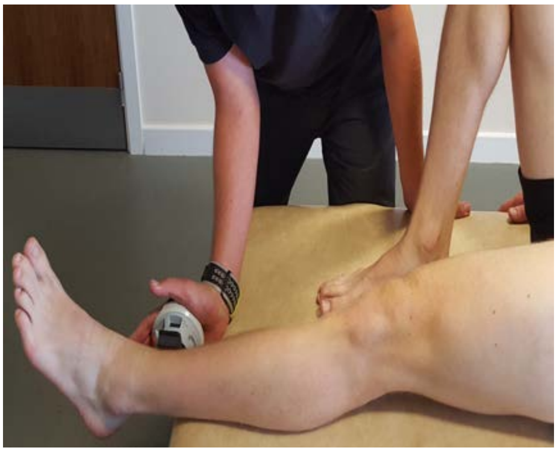

*(d)*

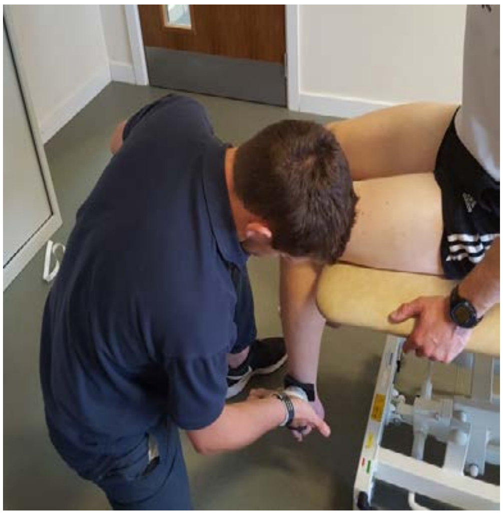

*(e)*

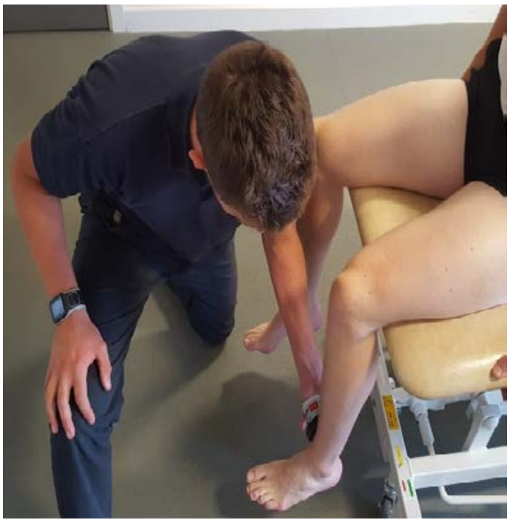

*(f)*

**Figure 1**. Participant positioning for strength testing of (a) flexion, (b) extension, (c) abduction, (d) adduction, (e) internal rotation, (f) external rotation. Position of the dynamometer is standardised in accordance with Thorborg et al, Scand J Med Sci Sports, 2010; 20: 493-50.
